# Efficacy and Safety of Immunotherapy for Cervical Cancer—A Systematic Review of Clinical Trials

**DOI:** 10.3390/cancers14020441

**Published:** 2022-01-17

**Authors:** Mona W. Schmidt, Marco J. Battista, Marcus Schmidt, Monique Garcia, Timo Siepmann, Annette Hasenburg, Katharina Anic

**Affiliations:** 1 Department of Gynecology and Obstetrics, University Medical Centre Mainz, Langenbeckstraße 1, 55131 Mainz, Germany; marco.battista@unimedizin-mainz.de (M.J.B.); marcus.schmidt@unimedizin-mainz.de (M.S.); annette.hasenburg@unimedizin-mainz.de (A.H.); katharina.anic@unimedizin-mainz.de (K.A.); 2 Division of Health Care Sciences Center for Clinical Research and Management Education Dresden, Dresden International University, 01067 Dresden, Germany; med.moniquegarcia@gmail.com (M.G.); timo.siepmann@uniklinikum-dresden.de (T.S.); 3 Department of Medicine, Pontifícia Universidade Católica de Minas Gerais (PUC MG), Betim 32604-115, Brazil; 4 Department of Neurology, University Hospital Carl Gustav Carus, Technische Universität Dresden, 01307 Dresden, Germany

**Keywords:** cervical cancer, immunotherapy, checkpoint inhibitors, vaccine, adoptive cell transfer therapy, PD-L1, CTLA-4, CAR T cells

## Abstract

**Simple Summary:**

Cervical cancer is the 4th leading cause of cancer deaths in women worldwide. Surgery, chemotherapy, radiotherapy and chemoradiation therapy are routinely used in the treatment of cervical cancer, while immunotherapy remains a novelty. The aim of our systematic review was to provide an extensive overview of the efficacy and safety of immunotherapy in cervical cancer patients. A total of 50 clinical trials assessed immune checkpoint inhibitors, therapeutic vaccines and adaptive cell transfer therapy. Overall, immunotherapy showed an acceptable safety profile. While the level of evidence on efficacy is still low, promising results, including few complete remissions in heavily pretreated women with metastatic disease, have been observed. Furthermore, a recent phase III trial assessing pembrolizumab in combination with chemotherapy (±bevacizumab) demonstrated a prolonged overall survival and has now led to a new standard of care for first-line systemic treatment in persistent, metastatic or recurrent cervical cancer patients.

**Abstract:**

Purpose: To systematically review the current body of evidence on the efficacy and safety of immunotherapy for cervical cancer (CC). Material and Methods: Medline, the Cochrane Central Register of Controlled Trials and Web of Science were searched for prospective trials assessing immunotherapy in CC patients in compliance with the Preferred Reporting Items for Systematic Reviews and Meta-Analyses guidelines. Full-text articles in English and German reporting outcomes of survival, response rates or safety were eligible. Results: Of 4655 screened studies, 51 were included (immune checkpoint inhibitors (ICI) *n*=20; therapeutic vaccines *n* = 25; adoptive cell transfer therapy *n*=9). Of these, one qualified as a phase III randomized controlled trial and demonstrated increased overall survival following treatment with pembrolizumab, chemotherapy and bevacizumab. A minority of studies included a control group (*n* = 7) or more than 50 patients (*n* = 15). Overall, response rates were low to moderate. No response to ICIs was seen in PD-L1 negative patients. However, few remarkable results were achieved in heavily pretreated patients. There were no safety concerns in any of the included studies. Conclusion: Strong evidence on the efficacy of strategies to treat recurrent or metastatic cervical cancer is currently limited to pembrolizumab in combination with chemotherapy and bevacizumab, which substantiates an urgent need for large confirmatory trials on alternative immunotherapies. Overall, there is sound evidence on the safety of immunotherapy in CC.

## 1. Introduction

Cervical cancer is the 4th most common cancer type in women and the most common gynecological tumor, accounting for around 342,000 deaths in 2020 [1]. Primary treatment options include surgery, (chemo)radiation therapy ((C)RT) or systemic chemotherapy (CHT) [2]. While great advances in preventing cervical cancer by prophylactic vaccinations have been achieved, systemic treatment options, especially for advanced, metastatic or recurrent cervical cancer, are still limited [3].

In 2017, the anti-angiogenesis drug Bevacizumab led to prolonged survival rates of around 3.5 months when combined with CHT [4]. It has since become the treatment of choice for primary therapy of persistent, metastatic or recurrent disease. However, 5-year recurrence rates remain high (28–73.6% for stage IIB-IVB), and patients being treated with 2nd line systemic therapy are faced with a mean overall survival time of around 7–9 months [5,6,7]. To date, no clear superiority of any ≥2nd line CHT or targeted therapy has been demonstrated upon recurrence [2]. Furthermore, substantial side effects of CHT need to be considered, especially in elderly multimorbid patients, and palliative care is a viable option that has to be discussed given the lack of effective systemic treatments in these patients.

With its success in the treatment of lung cancer, melanoma or renal cell carcinoma, immunotherapy has gained increasing popularity in recent years. The National Cancer Institute defines immunotherapy as a “type of therapy that uses substances to stimulate or suppress the immune system to help the body fight cancer, infection or disease” [8] and includes different approaches such as immune checkpoint inhibitors (ICI), adaptive cell transfer therapy (ACTT), therapeutic vaccines and immune system modulators. Overall, ICIs are currently the most prominent representatives of immunotherapy and are being investigated in numerous cancer types, including gynecological cancers [9]. However, as most cervical cancers are associated with the human papillomavirus (HPV), targeted immunotherapies such as therapeutic vaccines or ACTT are also emerging. While immunotherapy is already an integral part of therapy in some cancer types, tumor responses to immunotherapy can vary drastically between cancer types. Despite various promising approaches, immunotherapy is still at the beginning of being clinically explored for cervical cancer.

Thus, this systematic review aims to present the current clinical evidence on the efficacy and safety of immunotherapy in cervical cancer patients, mainly addressing the following questions: (1) Which immunotherapies have been clinically assessed in cervical cancer patients?; (2) Does cervical cancer respond to immunotherapy treatment?; (3) Does immunotherapy prolong survival in cervical cancer patients? As a secondary aim, the safety of immunotherapy in cervical cancer patients is evaluated.

## 2. Materials and Methods

This systematic review is reported in line with the Preferred Reporting Items for Systematic Reviews and Meta-Analyses (PRISMA) statement [10]. The systematic review was prospectively submitted to PROSPERO. Due to the automatic check currently performed by PROSPERO the submission was rejected just after finishing the manuscript because of an incorrectly filled question. Thus, a correction was not possible anymore and no registration number is available.

### 2.1. Literature Search

Three electronic bibliographical databases, MEDLINE (via Ovid), the Cochrane Central Register of Controlled Trials (CENTRAL) and Web of Science, were searched systematically without any restrictions towards language or publication date [11]. The creation and optimization of the search strategies for each database were aided by a librarian from Johannes-Gutenberg University Mainz. The search strategies were developed according to the following PICOS criteria [12]:

- P (patients/participants)—Adult patients with histologically proven cervical cancer;
- I (intervention)—Any form of immunotherapy;
- C (comparison)—Any (including chemotherapy, targeted therapy, surgery or placebo) or no comparison;
- O (outcome)—At least one measure of survival outcomes, response rates or adverse events;
- S (study design)—All types of prospective study designs.

All search strategies included index terms as well as free text related to cervical cancer and immunotherapy, including therapeutic vaccines, checkpoint inhibitors and CAR T cells. The search strategies are provided in the Supplementary Material (Appendix A). The search was performed on the 29th of September 2021. A cross-reference check was performed on all included studies by screening their reference lists and by using Google Scholar to identify articles that cite the included studies. Furthermore, studies included in related systematic reviews and meta-analyses were screened for eligibility. This was performed between the 23rd–30th of November 2021. Grey literature, including conference abstracts or commentaries, were not considered for the systematic review; however, highly relevant abstracts were included in the discussion if sufficient data was provided.

### 2.2. Eligibility Criteria

Original, prospective clinical trials (phase I-IV) published as complete journal articles were included. Eligibility criteria were based on the PICOS criteria reported above. Furthermore, only full-text articles written in English or German were included. Studies were excluded if (1) only a single patient with cervical cancer was reported (e.g., case reports, studies recruiting various cancer types), (2) a more recent publication of the same study was available (except when reporting different outcomes) or (3) due to a retrospective study design. As single-arm cohort trials are commonly used in phase I and II clinical trials in oncology and only a few comparative trials were expected in this emerging field, non-comparative trials were also included in this systematic review, despite the known increased risk of bias in these trials. Results were and should be interpreted accordingly.

### 2.3. Study Selection and Data Extraction

Title and abstract screening, as well as full-text screening, were conducted by two review authors (M.W.S. and K.A.) independently. Any disagreements over a particular study were resolved through discussion with a third reviewer (M.J.B.). Data extraction was performed by M.W.S. and re-checked independently by K.A. using predefined word spreadsheets, which were tested and adapted based on a few sample studies. Data extraction included an individual study identifier (author, title, and year of publication), fundamental study details including study population, study phase, programmed death ligand 1 (PD-L1) status for ICI trials and HPV status for therapeutic vaccines and ACTT, interventions and results. Efficacy data were extracted as survival and response data. Survival data included: (1) months and confidence interval (CI) for progression-free survival (PFS) and overall survival (OS), (2) (estimated) PFS or OS rates at 1 year and/or longest follow-up in percent, and (3) recurrence rates (%) for patients treated with curative intent. Response parameters included: (1) objective response rates (ORR, %), (2) disease control rates (OS, %) and (3) duration of response (DOR, months). Time to response was not extracted as initially planned, as it was reported by too few trials. When not provided, ORR (complete response + partial response) and DCR (complete response + partial response + stable disease) were calculated with available data. When possible, data for the subgroups based on PD-L1 status were reported separately.

Safety details of treatment-related adverse events (TRAE) were extracted as percentages based on the following: (1) treatment-related deaths (TRD), (2) TRAE of any grade (%), (3) TRAE grade 3 or higher (%), (4) list of TRAEs occurring in more than 5% (TRAEs≥ grade 3) or 10% (TRAEs of any grade), and (5) if available, the overall percentage of potentially immune-mediated AEs or the list of potentially immune-mediated AEs above 10% if the overall percentage was not given. If TRAEs were not reported, adverse events (AE) were described following the same rules.

### 2.4. Risk of Bias Assessment

Two review authors (M.W.S. and K.A.) independently assessed the risk of bias and study quality, and disputes were settled in discussion with a third reviewer (M.J.B). The revised Cochrane Risk of Bias tool for randomized trials (RoB-2) [13] was used for randomized control trials (RCTs). Unfortunately, no adequate risk of bias assessment tool with high validity evidence exists for single-arm cohort trials, which includes the majority of phase I and II trials in oncology, and available tools for comparative trials are not applicable to non-comparative trials. Thus, the risk of bias in single-arm cohort trials was assessed to judge the reliability of the outcome of each study based on domains assessed by the Risk of Bias in Non-Randomised Studies of Interventions tool (ROBINS-I tool) [14]. The risk of bias was judged as high, low or unclear. As no direct comparison between groups was made, the item “bias in selection of participants” was not evaluated. For bias due to confounding, factors were judged that may have an effect on the main outcomes, such as the use of immune-suppressants or other potential treatments upon the termination of trial treatments. Bias due to deviations from intended interventions was considered high for any other analysis than intention-to-treat, due to the possibility of overestimating the true efficacy benefit. To assess bias due to missing data, an availability of 90% (due to small sample sizes, one missing person can already be below the often used 95% threshold) of outcome data was regarded as sufficient. The risk of bias in measurement outcomes was judged based on the objectivity of efficacy or safety data, meaning the likelihood of misclassifying an outcome (e.g., stable vs. progressive disease in clinical examinations). Bias in the classification of interventions and bias in the selection of reported results was judged as written in the ROBINS-I tool. A similar approach of adapting the ROBINS-I tool was recently used in a Cochrane systematic review by Jullien et al. [15]. The risk of bias was assessed on the study level based on efficacy outcomes. For studies in which no adequate efficacy measurements were assessed, risk of bias judgment was done for clinical safety evaluations.

## 3. Results

### 3.1. Study Selection

**Figure 1:**
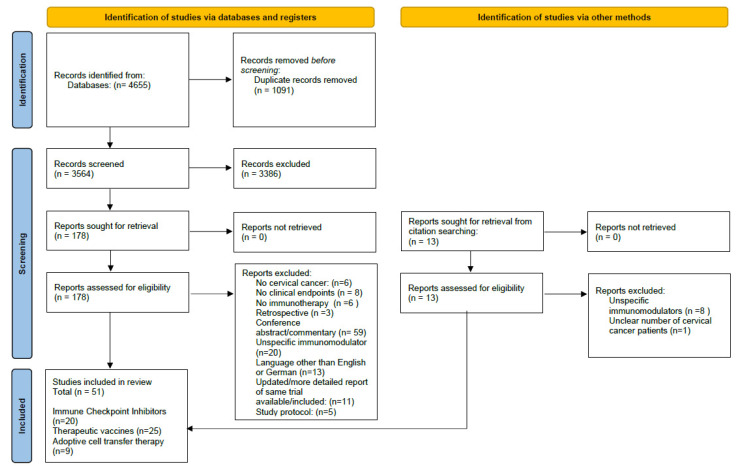
PRISMA flowchart of the screening and inclusion process.

Through the search, a total of 4655 studies were identified, of which 51 were included in the systematic review. A detailed flowchart of the study selection process is depicted in Figure 1.

### 3.2. Immune Checkpoint Inhibitors

ICIs block so-called “checkpoint” proteins, which are relevant in downregulating or inhibiting the immune response. Checkpoint proteins are expressed on T lymphocytes, antigen-presenting cells, and on many tumor cells, including HPV-associated tumors such as cervical cancer. While in physiological conditions, these checkpoint proteins are necessary to prevent the development of autoimmunity, cancer cells use them to evade immunosurveillance by overexpressing these checkpoint proteins. Commonly known checkpoints include PD-L1 on tumor cells as well as PD-1 or cytotoxic T-lymphocyte antigen 4 (CTLA-4) on T cells. However, new immune checkpoints such as idolamin-2,3-dioxygenase 1 (IDO1), a key component in tumor microenvironments, are currently being evaluated in clinical trials [16].

#### 3.2.1. Clinical Trials in Cervical Cancer

A total of 20 prospective clinical trials assessing the effects of checkpoint inhibitors were identified, of which 3 used PD-L1 inhibitors, 15 used PD-1 inhibitors, 2 used CTLA-4 inhibitors, 2 used IDO1 inhibitors and 1 study utilized anti-NKG2A antibodies (see Table 1 and Table 2). Trials were published between 2017 and 2021. Overall, pembrolizumab was the most researched agent (*n* = 7, [17,18,19,20,21,22,23]), and it is the only agent with published results of a phase III trial [17]. Other checkpoint inhibitors included nivolumab (*n* = 4, [24,25,26,27]), atezolizumab (*n* = 2, [28,29]), ipilimumab (*n* = 2, [30,31]), camrelizumab (*n* = 1, [32]), cemiplimab (*n* = 1, [33]), balstilimab (*n* = 1, [34]), bintrafusp alfa (*n* = 1, [35]), navoximod (*n* = 1, [29]) and monolizumab (*n* = 1, [36]), which is an anti-NKG2A (inhibitory receptor on natural killer cells) antibody. A total of 3 trials assessed a combination of immunotherapies from different subgroups (e.g., ICI plus vaccine) and are thus addressed and counted to both subgroups. A total of 15 studies focused on cervical cancer patients only or reported cervical cancer patients as a subpopulation in detail (Table 1). The remaining 6 studies included cervical cancer patients in the analysis of a broader population of advanced solid tumors (Table 2). Checkpoint inhibitors were evaluated as monotherapies in 10 studies, as well as in combination with various other treatments including chemotherapy (*n* = 2), radio(chemo)therapy (*n* = 3), targeted therapies (*n* = 3), therapeutic vaccines (*n* = 1), tumor-infiltrating lymphocytes (*n* = 1), adoptive T cell therapy (*n* = 1) and hypothermia (*n* = 1). An overview can be found in Table 1 and Table 2.

#### 3.2.2. Efficacy of Immune Checkpoint Inhibitors Based on PD-L1 Status in Cervical Cancer

Four studies with monotherapy of ICIs or placebo control reported subgroup analyses with regards to PD-L1 status in cervical cancer patients. Colombo et al. [17] reported a better HR with regards to PFS in PD-L1 positive patients treated with pembrolizumab plus chemotherapy ± bevacizumab compared to PD-L1 negative patients (CPS 1-<10% 0.68 [0.49–0.94] vs. CPS < 1% 0.94 [0.52–1.70]) with an even better effect in the PD-L1 highly positive group (CPS ≥10% 0.58 [0.44–0.77]), as well as for OS (CPS ≥ 10% 0.61 [0.44–0.84; CPS 1-<10% 0.67 [0.46–0.97]; CPS < 11.00 [0.53–1.89]). Overall, no survival benefit was seen in PD-L1 negative patients. Chung et al. demonstrated similar results in their phase II trial evaluating pembrolizumab monotherapy in patients who had progressed or were intolerant to standard-of-care systemic therapy. The ORR was 0% in the PD-L1 negative population (*n* = 15) compared to 14.6% [7.7–24.2] in the PD-L1 positive population [18]. Updated results were presented at the 52nd Annual Meeting of the Society of Gynecologic Oncology 2021 with 17 months of additional follow-up. The limited results available in the conference abstract showed a slight increase in the ORR in PD-L1 positive patients (17.1% [9.7–27.0]) and a DCR of 30.6% in the overall population [37]. In 2020, Tamura et al. reported similar results for the use of nivolumab monotherapy in advanced or recurrent cervical cancer with an ORR of 33% [80%-CI: 17–53] in PD-L1 positive patients (*n* = 15) compared to 0% [80%-CI 0–37] in PD-L1 negative patients (*n* = 5). Furthermore, 6-month OS was 86% [80%-CI: 69–94] in the PD-L1 positive group and 80% in PD-L1 negative patients [80%-CI: 45–94] [25]. On the contrary, a response rate of 7.9% in PD-L1 negative patients (*n* = 38%) was seen under balstilimab monotherapy in patients with metastatic, persistent or recurrent cervical cancer. However, a larger ORR of 20% was seen in PD-L1 positive patients (*n* = 58) [34].

#### 3.2.3. Efficacy and Safety of Checkpoint Inhibitors with or after (Chemo)Radiotherapy

When considering the use of ICIs in first-line therapy, their safety and efficacy combined with CRT are important to evaluate. Duska et al. conducted a phase II RCT to assess the safety of pembrolizumab during vs. after CRT in 52 locally advanced cervical cancer patients [20]. In their preliminary report, an overall similar and acceptable safety profile was demonstrated. The study is currently ongoing, and a follow-up including long-term safety data is expected. Mayadev et al. evaluated the use of 4 cycles of ipilimumab after CRT with an acceptable safety profile. Median PFS and OS were not reached within the 14.8-month follow-up period [30]. The combination of ICI and RT in patients resistant or intolerant to platinum and taxane doublet chemotherapy was assessed by Rischin et al. by administering cemiplimab with or without concurrent hypofractionated RT in a non-randomized controlled trial. Most included patients (90%) had received prior cancer-related RT as well as bevacizumab (70%). One patient died due to severe pneumonitis in the concurrent hypofractionated RT. In general, a similar safety profile was observed [33]. The overall response rates were comparable between both groups, with an increased DCR in the hypofractionated RT group (60% [95% CI 26.2–87.8] vs. 40% [95% CI [12.2–73.8]), but an increased median OS in the cemiblimab monotherapy group (10.3 months vs. 8.0 months) as well as an increased DOR (11.2 months vs. 6.4 months). However, the upper limit of the 95% CI was not reached in both groups.

### 3.3. Therapeutic Vaccines

Human papilloma virus (HPV) infections are known to play an important role in the etiology of the majority of cervical cancer cases. While prophylactic vaccinations against HPV are widely available and have been proven to be effective in preventing cervical cancer [38], they are unable to eliminate existing tumor cells and precursor lesions. Thus, there is still a major need for therapeutic vaccines which aim to evoke a durable and strong immune response eliminating cancer cells carrying special antigens. Tumor antigens can roughly be divided into tumor-associated antigens, which can also be found on healthy cells but are generally overexpressed in tumor cells, and tumor-specific antigens, such as oncogenic viral antigens, which are foreign to the healthy cell [39]. E6 and E7 antigens are known to cause HPV-associated neoplastic changes by allowing the uncontrolled progression of cell cycles into the S phase [40]. Because many cervical cancers are associated with HPV, these tumor-specific antigens present a promising target to provoke specific immune responses without increasing autoimmunity.

#### Clinical Vaccine Trials in Cervical Cancer

A total of 25 trials were identified assessing therapeutic vaccines in cervical cancer patients [21,41,42,43,44,45,46,47,48,49,50,51,52,53,54,55,56,57,58,59,60,61,62,63,64], of which 4 were not specific to cervical cancer but included a larger population with various cancers. The trials were published between 1989 and 2020. An overview can be found in Table 3. To date, no phase III trial has been published. Three trials assessed the combination of therapeutic vaccines with concomitant chemotherapy (carboplatin/paclitaxel q3w). Two of them administered the vaccinations two weeks after chemotherapy starting with the second cycle [43,51], while Basu et al. interrupted the vaccinations for five weekly cycles of cisplatin [62]. Only Youn et al. evaluated the combined use of ICIs (pembrolizumab) with a therapeutic vaccine [21]. Vaccines were mostly injected subcutaneously; however some were given intravenously, intradermally or intramuscularly. They ranged from single-shot doses, a predefined number of doses to unlimited doses, or repeated injections until disease progression. No deaths were reported in immediate relation to the vaccine therapies. Common treatment-related adverse events included injection site reactions such as swelling, redness, itching, pain as well as systemic reactions with fever or flu-like symptoms. Overall only a few grade III or IV toxicities occurred.

### 3.4. Adoptive T Cell Transfer Therapy

Another interesting approach is adoptive cell transfer therapy, such as the use of cytokine-induced killer cells (CIK) or T cell transfer. The two main approaches of adoptive T cell therapy include the use of tumor-infiltrating lymphocytes (TILs) and engineered T cell receptor (TCR)/chimeric antigen receptor (CAR) T cell therapy, for which tumor-specific autologous or allogenic T cells are grown ex vivo and reinfused for treatment [65]. TILs do not have to be modified as they are gained from tumor biopsies of patients and are thus expected to recognize tumor-associated antigens. They are grown in the laboratory with the help of interleukins before being reinfused. Patients usually have to undergo lymphodepletion by CHT or RT. CAR T cell therapy, on the other hand, is an example of genetically modified T cells to express CARs. They do not rely on major histocompatibility complexes (MHC) to present tumor antigens that are often downregulated in tumor cells [66] but can directly recognize surface antigens of tumor cells. Another alternative to CAR T cell therapy but with a similar approach is modifying the physiological T cell receptor (TCR) complex to recognize specific tumor antigens.

#### Clinical Trials in Cervical Cancer Patients

A total of 9 clinical trials evaluated adoptive cell transfer therapies using CIKs (*n* = 3, [23,67,68]), TILs (*n* = 3, [27,69,70]) or engineered TCR (*n* = 3, [71,72,73]) in a total of 209 cervical cancer patients between the years 2015–2021. An overview of all trials can be found in Table 4. Up to now, CAR T cell therapy has not been clinically assessed for cervical cancer. Three trials reported treatment with TCR in a broader cancer population, including cervical cancer [71,72,73], and one trial evaluated combined immunotherapy with TILs and anti-PD1 (nivolumab) [27]. In all trials, except for the combination with nivolumab, chemotherapy was given prior to or during adoptive cell therapy. Five studies assessing TCR-engineered T cells or TILs used cyclophosphamid and fludarabine, a non-myoablative chemotherapy [69,70,71,72,73]. The other two larger trials were designed as RCTs assessing CHT vs. CHT plus dentric cell-cytokine induced killer (DC-CIK) cell infusions [66] and CHT plus RT vs. CHT plus RT and CIK infusions [68]. While Chen et al. demonstrated significantly prolonged survival rates through the addition of DC-CIK ACTT, Li et al. did not find the same difference with CIK ACTT combined with CHT and RT. However, the study population differed between both trials.

Overall, adoptive cell transfer therapy was proven to be safe in cervical cancer patients, with most observed adverse events being due to the associated chemotherapies and high-dose interleukin treatments. No autoimmune reactions were observed.

### 3.5. Nonspecific Immune System Modulators/Immunomodulating Agents

Various nonspecific immune system modulators, including interferon α, interleukin 12, extract derived from agaricus blazei murill kyowa, mycobacterium tuberculosis (Z-100) or streptococcal preparations (OK-432) as well as sizofiran and thymopentin or cornyebacterium parvum have been studied for cervical cancer [74,75,76,77,78,79,80,81,82,83,84,85,86,87,88,89,90,91,92,93,94,95,96,97]. All agents affect the immune system in a general way at different levels. However, probably due to the rise of targeted immunotherapies, only a few large trials assessing nonspecific immune system modulators have been conducted after the year 2000. Unfortunately, the term immunotherapy (including variations of it) was not as commonly used to describe a treatment approach back then, and our search strategies were found to be inappropriate to adequately report the current evidence of nonspecific immune system modulators in this systematic review. Thus, the respective trials were excluded. Nevertheless, a sample of 25 trials can be found in Table S1.

### 3.6. Risk of Bias Assessment

Only one trial was judged to be at low risk of bias (Figures S1–S4). In RCTs, the risk of bias was mostly high due to a high number of patients lost to follow up or dropping out as well as the non-blinding of patients and outcome assessors. All non-comparative trials were judged as at least unclear risk of bias, as confounding factors were not reported or regarded in these trials. Furthermore, missing outcome data, as well as inappropriateness of recurrence or response detection in trials conducted around the year 2000 (only clinical examinations or x-rays), were the most common reasons for a high-risk judgment in non-comparative trials. One study reported two different groups with varying therapies, but no comparison was made between these groups [33]. The study was thus assessed as a non-comparative trial.

## 4. Discussion

Despite constant advances in cancer therapy, the prognosis of locally advanced, recurrent or metastasized cervical cancer patients remains unsatisfactory. With a better understanding of tumor immunology, including tumor mechanisms of resistance and avoidance to the host’s immune response, immunotherapy has become one of the most promising approaches in cancer treatment. The idea of increasing and targeting the body’s already occurring natural fight against aberrant cells seems simple, but finding the most effective approach is difficult.

The highest level of evidence for immunotherapy is currently available for ICIs (Table 1 and Table 2). Based on the positive results of phase I/II trials on pembrolizumab for cervical cancer, the FDA approved pembrolizumab as a monotherapy in patients with recurrent or metastatic PD-L1 positive disease in 2018. Due to the recently published phase III trials by Colombo et al. with a prolonged OS of around 8 months, pembrolizumab has now been approved in patients in combination with CHT and bevacizumab [17]. While this is currently the only phase III trial for ICIs in cervical cancer, many are ongoing, and results are eagerly awaited. Promising interim results of the phase III EMPOWER trial were presented at the European Society for Medical Oncology (ESMO) congress in 2021. Recurrent or metastatic cervical cancer patients who had progressed after platinum-based CHT were treated with either cemiplimab or the investigators’ choice of CHT. An interim analysis of 608 patients clearly favored cemiplimab treatment with regards to OS (12.0 vs. 8.5 months, *p* < 0.001), PFS and ORR [98]. In summary, the current evidence on ICI for cervical cancer is encouraging; however, ORR for ICI monotherapy in patients progressing after platinum-based chemotherapy is still low. In particular, the subgroup of PD-L1 negative patients does not seem to benefit from ICI in cervical cancer. Considering the ORR of around or less than 25% for ICI monotherapy in cervical cancer, several studies aim to identify prognostic factors to anticipate a favorable reaction to ICIs in cancer patients [99]. Alternative approaches try to increase response rates and to avoid acquired immune resistance by combining ICIs with other systemic therapies. For example, by combining a therapeutic vaccine with pembrolizumab (anti-PD-1), a remarkable ORR of 42% and a DCR of 58% was reached in advanced or recurrent HPV-positive cervical cancer patients [21]. Even higher response rates of 55% and a DCR of 82% were reported from the CLAP trial, treating patients with camrelizumab (anti-PD1) and apatinib (a tyrosine kinase inhibitor), despite more than 55% of the patients having had 2 prior lines of systemic chemotherapy [32]. Interim results of the CheckMate 358 study demonstrated the efficacy of 2 combinations of nivolumab (anti-PD1) and ipilimumab (anti-CTLA4) in patients with recurrent and metastatic cervical cancer. Results were presented at the 44th European Society for Medical Oncology (ESMO) congress in 2019 [100]. However, between-study comparisons should be interpreted with caution, and direct comparisons of treatment regimens are needed to prove the superiority of either combination. Another interesting approach to improve the efficacy of ICI is to increase the amount of PD-L1 on the cell surface of tumors. Recently, the extracellular plasminogen activator inhibitor type I (PAI-1) was found to be responsible for internalizing PD-L1, and targeting PAI-1 with a pharmacological inhibitor (tiplaxtinin) has led to increased PD-L1 expression on tumor cells in vivo and in vitro. Thus, a combination of ICI with tiplaxtinin seems promising and has shown first synergistic effects in a murine model of melanoma [101]. Overall, ICI treatment in cervical cancer proved to be safe with expected adverse events observed in other cancer types [102]. The occurrence of adverse events slightly increased with the combination of ICI and CHT [17]. Nevertheless, no increase in severe toxicities was observed in combinations with CHT or RT. Various trials assessing ICI are currently ongoing, including combinations with RT or CRT (nivolumab (NCT03298893/NCT03527264), atezolizumab (NCT03612791/NCT03612791), dostarlimab (NCT03833479), durvalumab (NCT03830866), pembrolizumab (NCT04221945/NCT02635360), as well as evaluating ICI for neoadjuvant CHT (pembrolizumab (NCT04238988)).

Despite major advances in the use of prophylactic HPV vaccines, therapeutic vaccines for HPV+ or HPV- cervical cancers are still at the beginning of their development and use in humans. This is clearly demonstrated by the limited amount of phase II trials, as described in this systematic review. Nevertheless, the concept of therapeutic vaccinations to fight cancer is of great interest and has shown promising results in many pre-clinical trials [103]. Due to the large proportion of HPV infections in cervical cancers, the HPV oncogenes E6 and E7 are the targets of the majority of the tested therapeutic vaccines for cervical cancer. Overall, therapeutic vaccinations have proven to be safe in numerous phase I and II clinical trials (Table 3). No major allergic reactions occurred in the here-reported trials. Even in combination with chemotherapy [43,51] or with PD-1 checkpoint inhibitors [21], therapeutic vaccinations did not lead to a notable increase in serious adverse events. Interestingly, the immunological T cell response was stronger when vaccinations were given during chemotherapy rather than post-chemotherapy. T cell reactivity around two weeks after chemotherapy was found to be increased after the second cycle of chemotherapy and subsequent ones, possibly due to the normalization of abnormally high tumor-promoting myeloid cell populations, which are initially higher in the presence of a large tumor burden [51]. Similar immunological changes in response to chemotherapy have been demonstrated in ovarian cancer patients [104,105]. Due to the lack of RCTs, no concluding statement on the efficacy of the currently tested therapeutic vaccines can be given. However, the majority of therapeutic vaccinations were able to demonstrate an immunological response in cervical cancer patients, which may prolong OS. Melief et al. reported high ORR and DCR of 43% and 86%, respectively, and found significantly improved OS in a group of 77 cervical cancer patients with strong (higher than the median) immunological vaccine responses compared to those with low (lower than the median) immunological vaccine responses (16.8 months vs. 11.2 months; *p* = 0.012) when treated with an HPV E6 and E7 peptide-based vaccine ± pegylated INFα in addition to chemotherapy [43]. A meta-analysis found the OS in similar patient populations (advanced, metastatic or recurrent cervical cancer) treated with chemotherapy alone to be around 10–12.8 months [106]. Promising interim results of a currently ongoing phase II trial assessing a triple combination of an HPV 16 E6/7 based vaccine, a tumor-targeting IL12 immunocytocine and bintrafusp alfa (a PD-L1 and TGF-ß inhibitor) in patients with advanced, previously treated cervical, anal, head and neck, vulvar and vaginal cancer (*n* = 25) were recently presented at the ASCO Annual Meeting 2021. The triple therapy led to an ORR of 55.6% with an ongoing response of 80% after 8 months of follow-up. All checkpoint-inhibitor-naïve patients (*n* = 6) are still alive [107]. To date, there is no data on the safety and efficacy of combining therapeutic vaccines with CRT. However, an ongoing trial IMMNUOCERV (NCT04580771) is currently assessing a liposomal HPV-16 E6/E7 multipeptide vaccine (PDS0101) combined with CRT in advanced cervical cancer patients. Overall, therapeutic vaccines are a growing and promising research trend in cancer therapy, with multiple ongoing clinical trials, especially for HPV-associated cancers, including head and neck cancers, cervical, vulva, vaginal or anal cancers [108].

ACTT can be an appealing alternative strategy to target tumor-specific antigens and has shown remarkable results with complete responses in some patients with breast cancer [109] or metastatic melanoma [110]. Presently, few clinical trials have been conducted in cervical cancer patients, although with promising results. A total of 3 phase I and II trials on ACTT demonstrated complete remissions in altogether 5 pretreated, metastatic cervical cancer patients ongoing at 15–67 months. However, ORR was still only around 28–33% in these relatively small study populations (a total of 30 patients in these 3 studies) [69,70,72]. A phase I trial with antigen receptor-engineered T cell therapy against HPV E7 showed anti-tumor efficacy even in cervical and other cancer patients pretreated with PD-1 based immunotherapy. The authors explained this through the contrasting mechanism of actions. While TCR-engineered T cells directly target the tumor, PD-L1 checkpoint inhibition acts by disinhibiting the physiological anti-tumor response [73]. Based on this rationale, one could expect a positive effect by combining adoptive cell therapy with anti PD1 immunotherapy. As shown by Yin et al., combining TILs with nivolumab led to a response rate of 25% even in PD-L1 negative patients [27]. However, whether this is due to the combination or the TILs alone cannot be determined. Whether or not the concurrent single cycle of lymphocyte-depleting CHT adds to the antitumor effects of the treatment is unclear. Although cyclophosphamide has demonstrated antitumoral effects in several malignancies, it is not used in cervical cancer treatment. However, its analog ifosfamide has shown low response rates of 15.7% in platinum-naïve and 11% in platinum-treated cervical cancer patients with short durations of response ranging from 1.8–3.1 months [111,112]. Other than using HPV-targeted ACCT, the use of CIK has been explored for cervical cancer. Chen et al. found a significantly prolonged OS (3 year OS rates: 56.4% vs. 80%) as well as decreased recurrence rates (3-year recurrence rates: 46.2% vs. 22.5%) in cervical cancer patients stage IIB-IV post-surgery when treated with DC-CIK in addition to CHT with cisplatin in an RCT. The tolerability of the treatment regimen was not reported [67]. On the other hand, Li et al. found no significant difference with regard to OS in a mostly pretreated advanced cervical cancer cohort (Stage IIA-IV) when treated with CIK in addition to RT and CHT in their RCT, despite a significantly increased short-term ORR after 1 month (88.6% with CIK vs. 68.9% control, *p* < 0.05). However, the randomization process was not blinded, leading to an overall high risk of bias in this trial [68]. One explanation for the differing results could be the lack of co-culturing of DC with the CIK by Li et al., as it was shown that the cytotoxic abilities of CIK can be enhanced by co-culturing and resulting stimulation by DC [113]. However, the effect of both CIK and DC-CIK was demonstrated, for example, in lung cancer [114], gastric cancer [115] and colon cancer [116]. Thus, further clinical trials are warranted in cervical cancer based on these promising results. Currently, ongoing trials include a multicenter phase II trial assessing the efficacy and safety of TIL ± pembrolizumab (NCT03108495), a phase II trial of CIK in addition to radiofrequency (NCT02490748), a phase II trial for T cell receptor gene therapy targeting HPV 16 E7 in HPV-associated cancers (NCT02858310) and a phase I trial on HPV-E6 specific TCR-T cells ± anti PD1 therapy (NCT03578406).

The major limitations of this systematic review include the small number of high-quality trials, especially the lack of RCTs, as well as the heterogeneity in study populations making direct comparisons of trial results unreliable, which is why no data synthesis has been performed. Furthermore, in older trials, no computer tomography scans were performed to assess response rates, but often clinical examinations and x-ray of the chest were used. Thus, the overall results presented here should be seen as guidance for future large clinical trials and provide an extensive overview of the current evidence of different immunotherapies. Furthermore, besides the immunotherapies reported here, nonspecific immunomodulators, including but not limited to herbal extracts, interleukin or cytokine therapy, can be used to modulate the immune response to fight cervical cancer. However, our search strategies were not able to reliably detect all of these trials. Thus, they were excluded as the risk of missing relevant trials was too high to achieve the standard of a systematic review. Nevertheless, we have supplied an exemplary overview of various immunomodulating agents tested in cervical cancer patients in the Table S1.

To promote the role of immunotherapy in cervical cancer, larger clinical trials are needed, and few are currently ongoing. In particular, therapeutic cancer vaccines have not yet been assessed in large clinical trials, despite the success of prophylactic vaccinations in cervical cancer. However, besides evaluating the efficacy of currently known drugs that have shown promising results in phase I and II trials, new approaches to modify the body’s immune response as well as to increase the responsiveness of tumor cells to immunotherapy need to be developed, as response rates to immunotherapy remain low. Promising strategies include the combination of targeted and untargeted immunotherapies as well as increasing the amount of immunotherapeutic target structures on tumors by inhibiting their destruction or potentially inducing their expression.

## 5. Conclusions

Immunotherapy in cervical cancer is on the uprise. The first results of high-quality trials on ICIs have led to the approval of pembrolizumab for cervical cancer as a monotherapy and, recently, in combination with CHT and bevacizumab by the FDA. These results have changed the standard of care for patients with persistent, recurrent or metastatic PD-L1-positive cervical cancer. On the other hand, no equivalent immunotherapy option is currently available for PD-L1-negative patients who do not profit from ICIs. Despite still being at the beginning of clinical testing, therapeutic vaccines and ACTT are promising options and have shown some spectacular remissions, even in heavily pretreated patients. However, the overall response rates remain low. Initial investigations demonstrated the potential of combining different immunotherapeutic approaches to increase effectiveness due to synergistic mechanisms of action. As expected, common side effects were immune-related, and overall, ICIs as well as therapeutic vaccines have proven to be safe and are generally well tolerated, even during combination therapy with RT, CHT or CRT. Similarly, ACTT has not led to treatment-related deaths; however, the preceding non-myeloablative CHT, in particular, causes an increased rate of severe adverse events. Thus, the use of ICIs in fragile, elderly patients should be considered carefully even in clinical trials. All things considered, further clinical trials are needed to verify the effects of immunotherapy as single agents or as combination therapies in larger cohorts.

## Figures and Tables

**Table 1:** Overview and results of clinical trials assessing immune checkpoint inhibitors in cervical cancer patients only.

Study/ Author	Drug	Study Phase	Number of Patients	Line of Therapy Disease	PD-L1 Status	Treatment	Survival Outcomes ^+^	Response Rates	Most Common TRAE/AE:
Keynote-826/ Colombo 2021 [17]	Pembrolizumab vs. placebo	III (RCT)	617	P/M/R, no prior CHT, no curative treatment available, 19.8% no prior therapy	CPS: <1 = 11.2% 1–10 = 37.4% >10 = 51.4%	Paclitaxel + platinum based CHT ±bevacizumab +pemroblizumab (200 mg) or placebo every three weeks for up to 35 cycles	Pembrolizumab vs. placebo		
							PFS = 10.4 [9.1–12.1] vs. 8.2 [6.4–8.4] PFS in PD-L1+ = 10.4 [9.7–12.3] vs. 8.2 [6.3–8.5] OS = 24.4 vs. 16.3–16.5 OS in PD-L1+ = NR e2y OS = 50.4% [43.8–56.6] vs. 40.4 [34.0–46.6]% e2y OS in PD-L1+ = 53% [46.0–59.4] vs. 41.7% [34.9–48.2]	ORR = 65.9% vs. 50.8% ORR in PD-L1+ = 68.1% vs. 50.2% DOR= 18.0 m vs. 10.4 m DOR PD-L1+ = 18.0 m vs. 10.4 m	TRD: 0.7% vs. 1.3% Any TRAE: 97.1% vs. 97.1%, alopcia 55.7 vs. 55.7%, anemia 48.5 vs. 42.7%, nausea 33.9 vs. 38.8%, diarrhea 24.8% vs. 18.8%, peripheral neuropathy 24.4% vs. 25.2% Grade ≥3 TRAE: any 68.4 vs. 64.1%, anemia 24.8 vs. 21.0%, neutropenia 12.1 vs. 9.4%, thrombocytopenia 6.8 vs. 3.9%, hypertension 6.5 vs. 7.4% Potentially immune-mediated AE: 33.9% vs. 15.2%
Keynote-158/Chung 2019 [18]	Pembrolizumab	II	98 10.2 [0.6–22.7]	R/M (II-IVB) progression or intolerance in ≥1 lines of standard therapy	Positive (CPS ≥ 1): 83.7% Negative (CPS < 1): 15.3%	Pembrolizumab 200 mg q3w for up to 2 years	PFS = 2.1 [2.0–2.2] PFS in PD-L1+ = 2.1 [2.1–2.3] OS = 9.4 [7.7–13.1] OS in PD-L1+ = 11 [9.1–14.1] e12m OS: 41.4% e12m OS in PD-L1+ = 47.3%	ORR = 12.2% [6.5–20.4] ORR in PD-L1+ = 14.6% [7.8–24.2] DCR = 30.6% [21.7–40.7] DCR in PD-L1+ = 32.9% [22.9–44.2] DOR = Not been reached [≥3.7–≥18.6 months]	TRD = None Any TRAE: 65.3%, hypothyroidism (10.2%), decreased appetite (9.2%), fatigue (9.2%), diarrhea (8.2%) Grade ≥3 TRAE: any event 12.2% Potentially immune-mediated AE: hypothyroidism 11.2%, hyperthyroidism 9.2%,
Keynote-028/Frenel et al., 2017 [19]	Pembrolizumab	Ib	24	M, PD-L1+, progressed on standard therapy or no adequate standard therapy available	Positive: 100%	Pembrolizumab 10 mg/kg q2w up to 2 years	PFS =: 2 [2,3] OS = 11 [4–15] 6 m OS = 67%: 12 m OS = 40%	ORR = 17% [5–37] DOR = 5.4 [4.1–7.5]	TRD = None Any TRAE: 75%, rash 21%, pyrexia 17% Grade ≥ 3 TRAE: any event 20.8%, rash 8% Potentially immune-mediated AE: 25%
Duska et al., 2020 [20]	Pembrolizumab	II (RCT)	52	A (IB-IVA), first line	Not reported	Pembrolizumab 200 mg q3w for 3 cycles during vs. after CRT (Cisplatin)	Pembrolizumab after vs. during CRT		
							Not reported	Not reported	TRD: None Grade ≥2 TRAE: 87.5% vs. 89.3%, nausea 45.8 vs. 41.6%, anemia 50% vs. 50%, decreased lymphocyte count 54.2% vs. 54.2%, decreased white blood cell count 41.7% vs. 54.2%, vomiting 29.2 vs. 16.7% Grade ≥3 TRAE: 62.5% vs. 67.9%, Lymphocytopenia 33.3% vs. 42.9%, leucopenia 16.7% vs. 25%, anemia 16.7% vs. 17.9%, neutropenia 16.7% vs. 10.7%, Potentially immune-mediated AE: 4.2% vs. 3.6%
Youn et al., 2020 [21]	Pembrolizumab (GY-188E vaccine)	II	36	A/R (inoperable) HPV-16+ or HPV-18+, progression with available therapies	Positive (CPS ≥ 1): 72% Negative (CPS < 1): 28%	Pemprolizumab 200 mg q3w for up to 2 years + GX-188E (vaccine) 2 mg i.m. at week 1, 2, 4, 7, 13, 19, 46	n = 26 PFS = 4.9 [2.1–6.7] OS = 10.2 [6.6–16.7]	n = 26 ORR = 42% [23–63] ORR in PD-L1+ = 50% [27–73] DCR = 58% [37–77] DCR in PD-L1+ = 65% [41–85] DOR = 4.0 [2.1–4.5]	TRD: None Any TRAE: 44%, hypothyroidism 11% Grade ≥ 3: any event 11% Potentially immune-mediated AE: 19%
NRG-GY002/Santin et al., 2020 [24]	Nivolumab	II	26	P/R/M, progression on systemic therapy	CPS ≥1 = 77.3% <1 = 22.7%	Nivolumab 3 mg/kg q2w for up to 46 doses	PFS = 3.5 [90% CI: 1.9–5.1] OS = 14.5 [90%CI: 8.3–26.8]	ORR = 4% [90%-CI: 0.4–22.9] DCR = 40% DOR = 3.8	TRD: None Any event: 96%, blood and lymphatic system disorder 56%, cardiac disorders 8%, endocrine disorders 24%, gastrointestinal disorders 80%, general disorders 72%, infections 28%, injury/poisoning/procedural complications 12%, investigations 52%, metabolism disorders 56%, musculoskeletal disorders 64%, neoplasms 8%, psychiatric disorders 20%, renal/urinary disorders 24%, reproductive system disorders 28%, respiratory/thoracic/mediastinal disorders 44%, skin disorders 28%, vascular disorders 36% Grade ≥ 3: any event 60%, blood and lymphatic system disorders 12%, gastrointestinal disorders 20%, investigations 20%, metabolism disorders 20%, neoplasms 8%
Tamura et al., 2019 [25]	Nivolumab	II	20	A/R (III-IV), progressed on ≥ 1 CHT	Positive (TPS ≥ 1) = 75% Negative (TPS < 1) = 25%	Nivolumab 240 mg q2w	PFS = 5.6 [80% CI: 2.8–7.1] OS = NE 6m OS = 84% [80% CI: 70–92%] 6m OS in PD-L1+= 86% [80% CI: 69–94]	ORR = 25% [80 CI: 13–41] DCR = 75% [80%-CI: 59–87] ORR in PD-L1+ = 33% [80% CI: 17–53] DOR = NE [80% CI: 3.0-NE]	TRD: None Any TRAE: 65%, increased AST/ALT 15/10%, hypothyroidism 15%, pruritus 15%, anemia 10%, arthralgia 10%, diarrhea 10%, pyrexia 10%, increased lipase 15%, malaise 10%, rash 20% Grade ≥ 3: any event 20%
Checkmate-358/Naumann et al., 2019 [26]	Nivolumab	I/II	19	M/R, ≥1st line	CPS ≥ 1% = 62.5% CPS < 1% = 37.5% Unknown: 15.8%	Nivolumab 240 mg q2w for up to 2 years	PFS = 5.1 [1.9–9.1] OS = 21.9 [15.1-NR] 12 m PFS = 26.3% [9.6–46.8] 12m OS = 77.5% [50.5–91.0] 24m OS = 49.8% [23.5–71.3]	ORR = 26.3% [9.1–51.2] DCR = 68.4% [43.3–87.4] DOR = NR (range: 23.3–29.5)	TRD: None Any TRAE: 63.2%, diarrhea 21.1%, fatigue 15.8%, pneumonitis 10.5%, abdominal pain 10.5%, stomatitis 10.5%, dry eye 10.5%, arthralgia 10.5%, skin disorders 21.1%, decreased appetite 5.3%, hepatocellulcar injury 5.3% Grade ≥ 3: any event 21.1%, diarrhea 5.3%, pneumonitis 5.3%, hepatocellular injury 5.3% Potentially immune-mediated AE: GI 21.1%, skin 21.1%, pulmonary 10.5%
Yin et al., 2020 [27]	Nivolumab (TIL)	I	80	M, progression after first-line CHT	Negative = 100%	Nivolumab 3 mg/kg q2w + TILs (average 50 × 10^9^)	PFS = 6.1 OS = 11.3	ORR= 25% DCR= 62.5%	TRD: None Any TRAE: 91.3%, fever 67.5%, fatigue 22.5%, rash 20%, anorexia 15% Grade ≥ 3: Any TRAE 5%, fever 5%
Mayadev et al., 2019 [30]	Ipilimumab	I	34	IB2-IVA LN+, first-line	Not reported	CRT followed by Ipilimumab 3 mg/kg q3w (4 cycles) or Ipilimumab 10 mg/kg q3w (4 cycles)	n = 21 PFS = not reached OS = not reached 12m OS = 90%	Not reported	TRD: None Any TREA: not reported Grade ≥ 3 TRAE: anemia 9.5%, GI disorders 9.5%, investigations 19%,
Lhereux et al., 2019 [31]	Ipilimumab	I/II	42	M, progression on ≥1 line platinum based CHT	Negative = 47.6% 1–10% = 9.5% >10% = 9.5%	Phase I: Ipilimumab 3 mg/kg q3w for 4 cycles Phase II: Ipilimumab 10 mg/kg q3w for 4 cycles	PFS = 2.5 [2.1–3.2] OS = 8.5 [3.6- not reached]	n = 34 ORR = 2.94%	TRD: None Any TRAE: fatigue 38.1%, diarrhea 29%, rash 29%, anemia 26.2%, nausea 26.2%, anorexia 23.8%, pruritus 21%, increase in ASAT/ALAT 21%, vomiting 16.7%, dehydration 11.9%, decreased lymphocyte count 16.7%, hypoalbuminemia 16.7%, hypomagnesemia 11.9% Grade ≥ 3 TRAE: anemia 9.5%, diarrhea 9.5%, hyponatremia 7.1% Potentially immune-mediated AE: diarrhea 29%, rash 29%, pruritus 21%, increase in ASAT/ALAT 21%, arthralgia 7%, peripheral neuropathy 5%, hypothyroidism 5%
Friedman et al., 2020 [28]	Atezolizumab	II	11	P/M/R, progression on ≥1 prior systemic therapies	Not reported	Atezolizumab 1200 mg q3w + bevacizumab 15 mg/kg q3w	PFS = 2.9 [1.8–6] OS = 8.9 [3.4–21.9] 1y OS = 36.4% [11.2–62.7%]	ORR = 0% DCR = 60%	TRD: None Any TRAE: hypertension 18%, diarrhea 27%, nausea 36%, ASAT/ALAT increase 27%/18%, gastrointestinal fistula 18%, fatigue 54%, fever 27%, dyspnea 185 Grade ≥ 3 TRAE: any 36.4%, thromboembolic event 9%, muscle weakness 9%, peripheral neuropathy 9%, arachnoiditis 9%, sensorineural hearing loss 9%, gastrointestinal bleeding 9%, anemia 9%, encephalopathy 9%, meningitis 9%
CLAP trial/Lan et al., 2020 [32]	Camrelizumab	II	45	P/M/R, progression on ≥1 prior systemic therapies	CPS ≥1 = 66.7%, CPS < 1 = 22.2%	Camrelizumab 200 mg q2w (maximum of 24 months) + apatinib 250 mg p.o. 1x/d	PFS = 8.8 [5.6-NE] PFS in PD-L1+ = sign. longer OS = NR [11.6-NE] 9m OS= 69.2% [52.9–80.8]	ORR = 55.6% [40.0–70.4] ORR in PD-L1+=69% DCR = 82.2% [67.9–92.0] DOR = NR [5.6-NE]	TRD: None Any TRAE: 95.6%, hypertension 84.4%, anemia 60%, proteinuria 55.6%, increased liver enzymes in up to 46.7%, fatigue 51.1%, Grade ≥ 3 TRAE: any 71.1%, hypertension 24.4%, anemia 20%, fatigue 15.6%, increased yGT 15.6%, neutropenia 6.7%, thrombocytopenia 6.6% Potentially immune-mediated AE: 33.3%
Rischin et al., 2020 [33]	Cemiplimab	I	10/10	M/R, Resistant or intolerant to platinum- plus taxane-based CHT	Not reported	Cemiplimab 3 mg/kg q2w for up to 48 weeks +/− hfRT	No hfRT: PFS = 1.9 [1.0–9.0] OS = 10.3 [2.1-NE] With hfRT: PFS = 3.6 [0.6–5.7] OS = 8.0 [1.7-NE]	No hfRT: ORR = 10% [0.3–44.5] DCR = 40% [12.2–73.8] DOR = 11.2 With hfRT: ORR = 10% [0.3–44.5] DCR = 60% [26.2–87.8] DOR = 6.4	No hfRT: TRD: none Any TRAE: 70%, fatigue 30%, diarrhea 20%, hypothyroidism 20%, pneumonitis 10%, hyponatremia 10%, myalgia 10% Grade ≥ 3 TRAE: any 10%, fatigue 10%, myalgia 10% With hfRT: TRD = 10% (pneumonitis) Any TRAE: 60%, fatigue 10%, diarrhea 30%, pneumonitis 20%, hyponatremia 10% Grade ≥ 3 TRAE: any 30%, pneumonitis 20%, hyponatremia 10%
O’Malley et al., 2021 [34]	Balstilimab	II	161	P/M/R, ≥1 platinum-based treatment regimen	CPS ≥ 1% = 61.5% CPS < 1% = 26.7%	Bastilimab 3 mg/kg q2w for up to 24 months	Will be reported separately	N = 140 ORR = 15% [10.0–21.8] ORR PD-L1+ = 20% DCR: 49.3% [41.1–57.5] DOR = 15.4 5.7-NR]	TRD: None Any TRAE: 71.4%, asthenia 23%, diarrhea 12.4%, pruritis 11.8%, fatigue 10.6% Grade ≥ 3 TRAE: any 11.8% Potentially immune-mediated AE: 6.8%

^+^ Reported in months [95% confidence intervals] if not otherwise indicated. Abbreviations: A: advanced; AE: adverse event, ALAT: alanine aminotransferase, ASAT: aspartate aminotransferase, CHT: chemotherapy, CPS: combined positive score, CRT: chemoradiotherapy, DCR: disease control rate; DOR: duration of response; eXy: estimated X year survival; hfRT: hypofractionated radiotherapy, HPV: human papillomavirus, M: metastatic, n: number of patients; NE: not estimable, NR, not reached, ORR: objective response rate; OS: overall survival; P: persistent, PD-L1: programmed cell death 1 ligand 1; PFS: progression-free survival; p.o.: per os, qXw; every X weeks, R: recurrent; RCT: randomized controlled trial, RT: radiotherapy, TPS: tumor proportion score, TRAE: treatment-related adverse events; TRD: treatment-related deaths.

**Table 2:** Overview and results of clinical trials assessing immune checkpoint inhibitors in patients with solid tumors, including cervical cancer patients.

Study/ Author/Year	Drug(s)	Study Phase	Number of Cervical Cancer Patients (All Patients)	Diseases Assessed	PD-L1/IDO1 Status	Treatment	Survival Outcomes ^+^	Response Rates	Most Common TRAEs:
Frumovitz et al., 2020 [22]	Pembrolizumab	II	6 (7)	Small cell neuroendocrine carcinoma, pretreated	Positive: 57.1% Negative: 28.6%	Pembrolizumab 200 mg q3w	PFS = 2.1 [0.8–3.2]	ORR = 0%	TRD: None Any TRAE: fatigue 29%, elevated ASAT/ALAT 29%, elevated alkaline phosphatase 14%, arthralgia 14%, rash 14% Grade ≥ 3 TRAE: any, elevated ASAT/ALAT 14%, elevated alkaline phosphatase 14%,
Qiao et al., 2019 [23]	Pembrolizumab (hypothermia; Adoptive cell therapy, chemotherapy))	I	4 (33)	Solid tumors, heavily pretreated	Not reported	All groups received 2 cycles hypothermia + 2 cycles adoptive cell transfer (CIK) Group 1: No additional therapy Group 2: + pembrolizumab Group 3: +chemotherapy	Not reported	ORR = Group 1: 30% Group 2: 27.30% Group 3: 30% DCR = Group 1: 70% Group 2: 55% Group 3: 75% ORR in cervical cancer patients = 25% DCR in cervical cancer patients = 75%	Of pembrolizumab group 2: TRD: None Any TRAE: rash 18.2%, subcutaneous fat induration 18.2%, diarrhea 18.2%, fatigue 18.2% Grade ≥ 3 TRAE: subcutaneous fat induration 9.1%
Jung et al., 2019 [29]	Atezolizumab + Navoximod	I	Dose escalation: 4(66) Dose expansion: 2(92)	A/M (incurable) solid tumors	Dose expansion: PD-L1+: 65.9% PD-L1-; 29.7% IDO-1+: 74.7% IDO-1-: 20.9%	Dose escalation: Atezolizumab 1200 mg q3w + navoximod (50–1000 mg twice daily per os) Dose expanding: Atezolizumab 1200 mg q3w + navoximod (600 or 1000 mg twice daily per os)	Not reported	Dose-escalation ORR = 9% ORR in PD-L1+ = 13% DCR = 26% Dose expansion: ORR = 11% ORR in PD-L1+ = 15% ORR in IDO1+ = 13%	TRD: One in prostate cancer Any TRAE: 75%, fatigue 22%, rash 22%, chromaturia 20%, decreased appetite 12%, nausea 12% Grade ≥ 3 TRAE: any 22%, rash 9%
Tinker et al., 2019 [36]	Monolizumab (CD94/NKG2A)	I	Dose ranging 1(18) Cohort expansion: 9 (40)	A/M/R gynaecological cancers, pretreated	Not reported	Dose ranging: 1/4/10 mg/kg q2w Cohort expansion: 10 mg/kg q2w	Not reported	Dose ranging: ORR = 0% DCR = 41.2% DOR = 3.4 months (1.4–5.5) Cohort expansion: ORR = 0% DCR = 18.4% DOR = 3.4 months (2.6–14.8)	TRD: None Any TRAE: not clearly reported Grade ≥ 3 TRAE: any 15.5% (non-hematologic), hematologic: anemia 15.5%, lymphopenia 19.0%, hypoalbuminemia 8.6%, alkaline phosphatase elevation 5.2%
Strauss et al., 2020 [35]	Bintrafusp alfa (TGF-ß and PD-L1 inhibitor)	I/II	Phase I: 25 (42) Phase II: 8 (16)	Phase I: heavily pretreated advanced solid tumors Phase II: Advanced HPV-associated cancers	Not reported	Phase I dose-escalation: Bintrafusp alfa 0.3–30 mg kg q2w Phase I cohort expansion and phase II: Bintrafusp alfa 1200 mg q2w	Overall analysis: PFS = 24.2 [22.4–46.4] OS = NR [8.6-NR] e12m OS = 58.8% [44.3–70.8] e18m OS = 51.4% [36.5–64.3]	Overall analysis ORR = 30.5% [19.2–43.9] DCR = 44.1% [31.2–57.6] DOR = 19.1 months [9.6–27.3]	TRD: None Any TRAE: any 83.1%, pruritus 25.4%, dermatitis 20.3%, keratoacanthoma 15.3%, hypothyroidism 15.3%, rash 15.3%, anemia 15.35, fatigue 11.9% Grade ≥ 3 TRAE: any 27.1%, anemia 6.8%, skin lesions 6.8%

^+^ Reported in months [95% confidence intervals] if not otherwise indicated. Abbreviations: A: advanced; ALAT: alanine aminotransferase, ASAT: aspartate aminotransferase, CHT: chemotherapy, CPS: combined positive score, CRT: chemoradiotherapy, DCR: disease control rate; DOR: duration of response; eXy: estimated X year survival; hfRT: hypofractionated radiotherapy, HPV: human papillomavirus, IDO-1: indoleamine 2,3-dioxygenase 1, M: metastatic, n: number of patients; NE: not estimable, NR, not reached, ORR: objective response rate; OS: overall survival; P: persistent, PD-L1: programmed cell death 1 ligand 1; PFS: progression-free survival; p.o.: per os, qXw; every X weeks, R: recurrent; RCT: randomized controlled trial, RT: radiotherapy, TGF: transforming growth factor, TPS: tumor proportion score, TRAE: treatment-related adverse events; TRD: treatment-related deaths.

**Table 3:** Overview and results of clinical trials assessing therapeutic vaccines in cervical cancer patients.

Study/ Author/ Year	Name of Vaccine/ Antigen	Study Phase	Number of Cervical Cancer Patients (All Patients)	Stage of Cervical Cancer	HPV Status	Treatment	Survival Outcomes ^+^	Response Rates	Most Common TRAEs/AEs
Peptide-based									
Hasegawa et al., 2018 [41]	FOXM1, MELK, HJURP, VEGFR1/2	I	9	P/R HLA-A*2402	Not reported	0.5/1/2 mg of each peptide q1wk for up to 16 weeks, then q2w	PFS = 3.3	ORR = 0% DCR = 77.8% DOR = 1.7–10.3 months	TRD: None, Any AE: injection site reactions 66.7%, anemia 66.7%, increased creatinine 44.4%, vaginal hemorrhage 33.3% Grade ≥ 3 AE: anemia 11.1%
Kenter et al., 2008 [42]	HPV16 E6/E7	I	43	A/R, no options for further treatment	Not reported	s.c. q3w 4 times 3 groups with different doses and combinations	6 patients alive at 18–36 months, 4 of them received additional chemotherapy	1 CR at 36 months (no additional chemotherapy received), 5 SD at 18–26 months	TRD: None Any AE: injection site reactions 100%, fever 14.3%, flu-like symptoms 20% Grade ≥3 AE: 0%
Melief et al., 2020 [43]	ISA101 HPV 16 E6/E7	I/II	77	IIIB-IVA/M/R HPV 16+	HPV16+: 100%	2 weeks after 2nd, 3rd, 4th cycle carboplatin/paclitaxel, 4 different doses ± pegylated INFα	OS in strong vaccine response = 16.8 months OS in low vaccine response = 11.2 (*p* = 0.012, HR 0.491)	ORR = 43% DCR = 86% DOR = 5.2 months [3.5–6.9]	TRD. None Any TRAE: 98.9%, injection site reactions 69.4% Grade ≥3 TRAE: 86.3%
Reuschenbach et al., 2016 * [44]	VicOryx P16^INK4a^	I/IIa	17 (26)	IV M cancers, HPV+, overexpressing p16INK4a	Not reported	s.c. q1w for 4 doses then 1 week rest, up to 12 doses	PFS = 3.5 OS = 11.9	ORR = 0% DCR = 64%	TRD: None Any TRAE: mild injection site reactions 38.5%, rest not clearly reported Grade ≥3 TRAE: 0%
Steller et al., 1998 [45]	HPV 16 E7	I	12	P/R (IB1-IVA), not amenable to surgery or radiation therapy HLA-A2+	HPV 16+: 75%	s.c. q3w for 4 doses, 4 different dose escalation groups	2 SD at 6 and 3 months, 6 alive with PD at 2–7 months	ORR = 0%	TRD: None Any TRAE: mild injection site reactions, not clearly reported Grade ≥3 TRAE: not clearly reported
Takeuchi et al., 2020 * [46]	URLC10/ HIG-2/FOYM1, MELK, HJURP	I/II	Phase I: 11 (23) Phase II: 20 (66)	P/R cervical or ovarian cancer, median 3–5 prior therapies, HLA-A*0201 orA*2402	Not reported	s.c. q1w for 12 doses, followed by q2w for 8 doses, followed by q4w (after 1 year by choice q1m, q3m or q4m)	Cervical cancer *n* = 15: Physical state and treatment-related dermatological reactions (3.3 vs. 21.2 months, HR 6.4 [1.38–29.24] were strongly associated with prolonged OS.	Cervical cancer *n* = 15: ORR = 20% DCR = 80%	Whole population *n* = 64: TRD: None Any TRAE: injection site reactions Grade ≥ 3 TRAE: injection site ulceration 7.8%, lymphocytopenia 15.6%
Tsuda et al., 2004 * [47]	Different peptides	I	7 (14)	Ib-IVA Gynecologic cancer HLA-A2 or A24 +	Not reported	s.c.3 injections q2w, followed by 1 injection q2w	Not reported	Cervical cancer: ORR = 18.6% DCR = 57.1%	TRD: None Any TRAE: fever 31.4%, dermatologic 57.1% Grade ≥ 3 TRAE: 7.1%
Van Driel et al., 1999 [48]	HPV16 E7	I-II	19	IA-IVB P/R, not amenable to other treatments HPV 16+, HLA-A*201+	HPV 16+: 100%	s.c. q3w for 4 doses, dose-escalation	OS = 7 (range: 0–22)	ORR = 0% DCR = 21.1%	TRD: None Any TRAE: Mild injection site reactions 21.1%, induration 10.5%, lymphocytopenia 57.9% Grade ≥ 3 TRAE: not clearly reported
Van Poelgeest et al., 2013 * [49]	HPV16 E6/E7	II	17 (20)	A/R gynecological carcinoma HPV 16 + No curative treatment options	HPV 16	s.c. q3w for 4 doses	Cervical cancer: OS = 8.8 (range 4–37)	All patients: ORR = 0% DCR = 27.3% DCR including non-target lesions= 0%	Overall population: TRD: none Any TRAE: Injection site reaction 100%, fever 40%. Chills 30%, fatigue 20%, nausea 30%, flu-like symptoms 35% Grade ≥ 3 TRAE: 0%
Welters et al., 2008 [50]	HPV 16 E6/E7	II	6	Resected IB1 HPV 16+	HPV 16+: 100%	s.c. q3w for 4 doses	3 patients free of disease at 10/13/24 months, 2 recurrences at 7 months after last vaccination and at the time of 3rd vaccination	RR= 33.3%	TRD: None Any TRAE: mild pain 100%, fever 50%, flu-like symptoms 50%, injection site reactions 100% Grade ≥3 TRAE: 0%
Welters et al., 2016 [51]	HPV 16 E6/E7	I	13	A/M/R	HPV 16+: 66.7%	Two weeks after second or third cycle of CHT (Carboplatin/Paclitaxel)	Not reported	Not reported	TRD: None Any AE: 58.3%, injection site reactions, fever Grade ≥ 3 TRAE (vaccine): 8%
Cell-based (Dendritic cell/B-cell-Monocyte)									
Choi et al., 2020 [52]	BVAC-C HPV 16/18 E6/E7	I	11	M, progressed after platinum-based chemotherapy, HPV16/18+	HPV 16+: 82% HPV 18+: 18%	i.v. injection q4w for 3 cycles	PFS = 6.8 [3.2-NR] OS = 12.0 [12-NR] 12mOS = 65% [39–100]	ORR = 11% [0–32] DCR = 67%	TRD: None Any TAE: pyrexia 55%, myalgia 36% Grade ≥ 3 TRAE: 0%
Ferrara et al., 2003 [53]	HPV 16/18 E7	I	15	P/R, with no other therapy option, HPV 16/18+	HPV16 +: 80% HPV 18+: 20%	s.c. injection every 10–21 days	Not reported	ORR = 0% DCR = 0%	TRD. None No clearly vaccination related AE
Rahma et al., 2014 [54]	HPV 16 E6/E7	I	18 (E6) 14 (E7)	A/P/R HPV 16 or 18+	HPV 16+: 56.3% HPV 18+: 43.8%	i.v. q3w for 2 cycles, the q4w (maximum of 14 vaccinations)	PFS = 3.5 OS = 10.0	ORR = 0%	TRD: None Any AE: Not clearly reported, fatigue 56.3% Grade ≥ 3 AE: No grade 3 events ≥ 5%
Ramanathan et al., 2014 [55]	Primed by tumor RNA/tumor lysate/cervical cancer cell line	I (RCT)	14	R (after initial radical treatment) HPV+	Not reported	i.d. q2w 3 times Group I: saline control Group II: unprimed matured DC Group III: primed mature DC	Not clearly reported 1 alive and disease free after additional CHT after 8 years	Not clearly reported	TRD: None Any grade: 21.4%, fever 14.2%, itching 7.1%, UTI 7.1%, elevated bilirubin and alkaline phosphatase 7.1% Grade ≥ 3: None Itching, fever, vomiting
Santin et al., 2006 [56]	HPV 16/18 E7	I	4	P/R, No other treatment option, HPV 16/18 positive	HPV 16+: 25% HPV 18+: 75%	s.c. q2w for 5 doses followed by q30d for 5 doses, followed by q60d for 3 doses, each with twice daily IL-2 from day 3 to7 post-vaccination	2 patients died after 5 months, 2 after 13 months	ORR = 0%	TRD: None Any TRAE: injection site reactions 50%, flu-like symptoms 100%, draining lymph node enlargement 50% Grade ≥ 3 TRAE: Not clearly reported
Santin et al., 2008 [57]	HPV 16/18 E7	I	10	IB after rad. Hysterectomy, HPV 16 or 18 +	HPV 16+: 90% HPV 18+: 10%	s.c. q3w for 5 doses, dose escalation 3–4 patients per dose	All patients alive after 17–31 months	RR = 0% (follow-up time 17–31 months)	TRD: None Any TRAE: not clearly reported, mild but increasing injection site reactions, draining lymph node enlargement Grade ≥ 3 TRAE: Not clearly reported
DNA-based									
HPV-004/Hasan et al., 2020 [58]	MEDI0456 (INO-3112) HPV 16/18 E6/E7	I/IIa	Cohort 1: 7 Cohort 2: 3	Cohort 1: new, inoperable stage IB-IVB Cohort 2: persistent or recurrent cancer, All: HPV 16/18 +, after CRT	HPV 16+: 70% HPV 18+: 30%	i.m. injection of 6 mg VGX-3100 and 1 mg INO-9012 followed by electroporation q4w for up to 4 doses	Cohort 1: PFS = NR e1y PFS = 100% Cohort 2: PFS = NR e1y PFS = 50%	Cohort 1 ORR = 100% Cohort 2 ORR = At least 33%	TRD: None TRAE: 80%, injection site bruising 20% and pain 20%. Grade ≥ 3 TRAE: 0%
Hui et al., 1997 [59]	HLA-A2/HLA-B/H-2K^k^-	II	3 (10)	M, refractory to all available therapies	Not reported	Injections. in cutaneous metastases q1w for four doses	Not reported	Cervical cancer 1 CR, 1 PR of injected cutaneous metastasis. All had systemic PD	TRD: None Any TRAE: Not adequately reported, no changes in hematological or liver function values Grade ≥ 3 TRAE:
Youn et al., * 2020 [21]	GY-188E HPV DNA E6/E7	II	26	A/R (inoperable) HPV-16+ or HPV-18+, progression with available therapies	Positive: 72% Negative: 28%	Pemprolizumab 200 mg q3w for up to 2 years + GX-188E (vaccine) 2 mg i.m. at week 1,2,4,7,13,19,46	n = 26 PFS = 4.9 [2.1–6.7] OS = 10.2 [6.6–16.7]	n = 26 ORR = 42% [23–63] ORR in PD-L1+= 50% [27–73] DCR = 58% [37–77] DCR in PD-L1+= 65% [41–85] DOR = 4.0 months [2.1–4.5] TTR = 2.1 months [2.1–3.0]	TRD: None Any TRAE: 44%, hypothyroidism 11% Grade ≥ 3: any event 11% Potentially immune-mediated AE: 19%
Virus-based									
Borysiewicz et. al. 1996 [60]	TA-HPV HPV 16/18 E6/E7	I/II	8	A/R, immunocompetent	HPV 16+: 100%	Single dose	6 out of 8 patients died within 2–14 months post vaccination, 2 were alive after 15 months (recurrent) and 21 months (Stage Ib) post-vaccination	Two patients were tumor free at 15/21 months post vaccination	TRD: Not clearly reported Any TRAE: injection site reactions 100% Grade ≥ 3 TRAE: Not clearly reported, no serious TRAEs
Freedmann et al., 1989 [61]	Viral oncolysate	II (RCT)	75	A (Lymph node metastases or large volume tumor) (No prior CHT or RT)	Not reported	RT ± i.d. viral oncolysate q1w RT then q2w for 12 months	PFS= 22.3 (RT + Viral oncolysate) vs. 15.1 months (RT) OS= 30.0 vs. 27.8 months	Not reported	TRD: None Any grade: delayed-type hypersensitivity reactions, chills and malaise, arthralgia Grade ≥ 3: Not reported Potentially immune-mediated AE: paraneoplastic syndrome 1.3%
Bacterial-based									
Basu et al., 2018 [62]	ADXS11-001 HPV 16 E7	II (RCT)	110	P/R (Prior CHT/RT/RT)	HPV 16+: 73.4% HPV 18+: 15.6%	Monotherapy: i.v. ADXS11-001 d1 + d29 + d57 vs Combination with Cisplatin: ADXS11-001 d1, followed by 5 doses cisplatin q1w after 4 weeks, followed by 3x ADXS11-001	ADXS11-001 monotherapy vs. ADXS11-001 + CHT		
							*n* = 69 PFS = 6.1 [5.9-.4] vs. 6.4 [4.2–8.9] OS = 8.3 [5.6–10.5] vs. 8.8 [7.4–13.3] 12m OS = 30.9% vs. 38.9%	*n* = 69 ORR = 17.1% vs. 15.7% DCR = 62.9% vs. 58.8% DOR = 7.2 months vs. 9.4 months (excluding SD)	TRD: None Any grade AE: 87.3% vs. 88.9%, possibly drug related: chills 30.9 vs. 35.2, pyrexia 12.7 vs. 13.0, nausea 5.5 vs. 3.7, vomiting 5.5 vs. 7.4% Grade ≥ 3 AE: any 22.2% vs. 18%
GOG-0265/ Huh et al., 2020 [63]	ADXS11-001 HPV 16 E7	II	54	M pretreated	Not reported	i.v. q4w	PFS = 2.8 [2.6–3.0] OS = 6.1 [4.3–12.1] 12m OS: 38%	ORR = 6% DCR = 16%	TRD: None Any TRAE: 98%, chills 58%, fatigue 54%, fever 36%, headache 36%, nausea 32% Grade ≥ 3 TRAE: 42%, anemia 10%, hypotension 12%, cytokine release syndrome: 12%
Maciag et al., 2009 [64]	ADXS11-001 HPV 16 E7	I	15	A/M/R, pretreated	HPV 16 positive: 66.7% HPV 18+: 0%	i.v. q3w 2 times, 3 groups with different dosing	OS = 347 days 11 died (median 281 days, IQR 118–367), 3 alive at 707–838 days	ORR = 7.7% (unconfirmed response) DCR = 61.5%	TRD: None Any AE: pyrexia 100%, vomiting 60%, chills 53.3%, headache 53.3%, anemia 53.3% Grade ≥3 TRAE: any 40%, pyrexia 20%, increased liver enzymes 13.3%, fatigue 6.7%

^+^ Reported in months [95% confidence intervals] if not otherwise indicated. * Trials included heterogeneous study populations, including various types of cancers. Abbreviations: A: advanced; AE: adverse event, CHT: chemotherapy, CR: complete response, CRT: chemoradiotherapy, DCR: disease control rate; DNA: desoxyribonucleic acid, DOR: duration of response; e1y: estimated 1 year survival; FOXM1: forkhead box M1, HIG-2: hypoxia-inducible gene 2, HJURP: holiday junction-recognition protein, HLA: human leukocyte antigen, HPV: human papillomavirus, HR: hazard ratio, i.d.: intradermal, i.m.; intramuscular, i.v.: intravenous, M: metastatic, MELK: maternal embryonic leucine zipper kinase, n: number of patients; NR, not reached, ORR: objective response rate; OS: overall survival; P: persistent, PD: progressive disease; PFS: progression-free survival; PR: partial response, qXw; every X weeks, R: recurrent; RCT: randomized controlled trial, RNA: ribonucleic acid, RR: recurrence rate, RT: radiotherapy, s.c.: subcutaneously; TRAE: treatment-related adverse events; TRD: treatment-related deaths, URLC10: upregulating lung cancer 10 gene, VEGFR ½: vascular endothelial growth factor receptors 1/2.

**Table 4:** Overview and results of clinical trials assessing adoptive T cell therapy in cervical cancer patients.

Study/ Author/Year	Type	Antigen	Adjuvant Chemotherapy	Study Phase	Number of Cervical Cancer Patients (All Patients)	Stage of Cervical Cancer	HPV	Treatment	Survival Outcomes ^+^	Response Rates	Most Common Adverse Events
Lu et al., 2017 * [72]	TCR	MAGE-A3	Cyclophosphamid + fludarabine	I	3 (17)	M (recurrent) HLA-DPB1*0401 + > 50% MAGE-A + tumor cells	Not reported	Chemotherapy daily for 5 days followed by single-dose T-cell infusion and IL-2	Not reported	Cervical cancer ORR = 33%	Overall population: TRD: None Any AE: 100%, prolonged fever after infusion 58.8% Grade ≥ 3 AE: any 100%, elevated liver enzymes 11.8%, elevated creatinine 11.8%, hypoxia 5.9%, dyspnoea 5.9%, atrial fibrillation 5.9%, renal failure 5.9%, confusion 5.9%
Nagarsheth et al., 2021 * [73]	TCR	HPV E7	Cyclophosphamid + fludarabine	I	5 (12)	M, HPV-associated epithelial cancers, pretreated	Not reported	Chemotherapy daily for 5 days followed by single-dose T-cell infusion and aldesleukin	No reported	Cervical cancer: ORR =: 40% DCR = 60%	Overall population: TRD: none Any AE = 100%, hematologic disorders 100%, electrolyte disorders 91.7%, fever 91.7%, fatigue 83.3%, diarrhea 83.3% Grade ≥ 3 AE: any 100%, hematologic disorders 100%, febrile neutropenia 66.7%, electrolyte disorders 66.7%, fever 8.3%, pulmonary disorders 33.3%, hypertension 8.3%, hypotension 16.6%, increased liver enzymes 8.3%, acute kidney injury 8.3%, weakness 8.3%, soft tissue necrosis, peripheral ischemia 8.3%
Doran et al., 2019 * [71]	TCR	HPV 16 E6	Cyclophosphamid + fludarabine	I/II	6 (12)	M HPV 16+ epithelial cancers, pretreated with platinum-based CHT	HPV 16+: 100%	Chemotherapy daily for 5 days followed by single-dose T-cell infusion and aldesleukin	Not reported	Cervical cancer: ORR= 0% DCR= 33.3% DOR = 4- 6 months	Overall population: TRD: None Any AE: 100% Grade ≥ 3 AE: hematologic disorders 100%, febrile neutropenia 38%, infection 31%, diarrhea 8%, rash 8%, pulmonary disorders 8%, syncope 8%, hyperbilirubinemia 8%
Stevanovic et al., 2015 [69]	TIL	Selected for HPV E6/E7 reactivity	Cyclophosphamid + fludarabine	I	9	M, pretreated	HPV 16: 22.2% HPV 18: 77.8%	Chemotherapy daily for 5 days followed by single-dose T-cell infusion and aldesleukin	Not reported	ORR= 33.3% DOR= 3 months for PR, ongoing at 15 and 22 months for CR	TRD: None Any AE: 100% Grade ≥ 3 A: 100%, anemia, hematological disorders 100%, infection 66.7%, febrile neutropenia 55.5%, metabolic disorders 55.5%, nausea/vomiting 44.4%, fatigue 33.3%, diarrhea 22.2%, hypoxia 22.2%, syncope/hypotension/hemorrhage/urethral obstruction 11.1% each
Stevanovic et al., 2019 * [70]	TIL	Selected for HPV E6/E7 reactivity	Cyclophosphamid + fludarabine	II	18 (29)	M, pretreated	HPV 16+: 27.7% HPV 18+: 61.1%	Chemotherapy daily for 5 days followed by single-dose T-cell infusion and aldesleukin	Not reported	Cervical Cancer: ORR= 28% DOR= 3 months in PR, ongoing at 53 and 67 months for CR	Overall population: TRD: None Any AE: 100% Grade ≥ 3 AE: 100%, hematologic disorders 100%, infection 58.6%, febrile neutropenia 41.4%, metabolic disorders 41.4%, hypoxia 27.6%, nausea/vomiting 20.7%, dyspnea 13.8%, diarrhea 10.3%, fatigue 10.3%, hypotension 10.3%, cystitis 6.9%, hemorrhage 6.9%, oliguria 6.8%, renal failure 6.8%, syncope 6.8%, urethral obstruction 6.8%
Yin et al., 2020 [27]	TIL		Nivolumab	I	80	M, persistent during 1st line CHT	Positive: 85% Negative: 15%	Nivolumab 3 mg/kg q2w + TILs (average 50 × 109)	PFS = 6.1 OS = 11.3	ORR = 25% DCR = 62.5% DOR= 12.8 months	TRD: None Any TRAE: 91.3%, fever 67.5%, fatigue 22.5%, rash 20%, anorexia 15%, leucopenia 6.3% Grade ≥3 TRAE: any 5%, fever 5%
Chen et al., 2015 [67]	DC-CIK		Cisplatin	II (RCT)	79	IIa-IV (prior treatment unclear)	Not reported	Interven.: Cisplatin 20 mg/d day 2–10 + reinfusion of DC-CIK after CHT Control: Cisplatin 20 mg/d for 10 days Both treatments were repeated after 3 months	CHT + DC-CIK vs. CHT only		
									1y RR = 5% vs. 28.2% 3y RR = 22.5% vs. 46.2 1y OS = 97.3% vs. 92.3% 3y OS = 80% vs. 56.4% (*p* < 0.005%)	Not reported	Not reported
Li et al., 2019 [68]	CIK		Paclitaxel or gemcitabine + cisplatin+ RT	II (RCT)	89	IIA-IV (40.1% had prior surgery or RT/CHT in the past 6 months)	Not reported	RT + CHT q4w± i.v. CIK once per day for 4 days followed by CHT (alternating for 4–6 courses)	CHT + RT + CIK vs. CHT + RT		
									1y OS = 93.2% vs. 88.9% 3y OS = 47.7 vs. 42.2% (*p* > 0.05)	ORR = 88.6% vs. 68.9% (*p* < 0.05)	TRD: None Any grade AE: Not reported, transient hypothermia 34.1% after CIK infusion
Qiao et al., 2019 * [23]	CIK	I	Hypothermia ± CHT ± pembrolizumab		4 (33)	Advanced solid tumors, heavily pretreated	Not reported	All groups received 2 cycles hypothermia + 2 cycles adoptive cell transfer (CIK) Group 1: No additional therapy Group 2: +pembrolizumab Group 3: +chemotherapy	Not reported	ORR = Group 1: 30% Group 2: 27.30% Group 3: 30% DCR= Group 1: 70% Group 2: 55% Group 3: 75% ORR in cervical cancer patients = 25% DCR in cervical cancer patients = 75%	Of pembrolizumab group 2: TRD: None Any TRAE: rash 18.2%, subcutaneous fat induration 18.2%, diarrhea 18.2%, fatigue 18.2% Grade ≥ 3 TRAE: subcutaneous fat induration 9.1%

^+^ Reported in months [95% confidence intervals] if not otherwise indicated. * Trials included heterogeneous study populations, including various types of cancers. Abbreviations: A: advanced; AE: adverse event, CHT: chemotherapy, CIK: cytokine-induced killer cells, CR: complete response, DC: dendritic cells, DCR: disease control rate; DOR: duration of response; eXy: estimated X year survival; HLA: human leukocyte antigen, HPV: human papillomavirus, IL-2: interleukine-2 M: metastatic, n: number of patients; NR, not reached, ORR: objective response rate; OS: overall survival; P: persistent, PFS: progression-free survival; PR: partial response, qXw: every X weeks, R: recurrent; RCT: randomized controlled trial, RR: recurrence rate, RT: radiotherapy, TCR: T cell receptor, TIL: tumor-infiltrating lymphocytes, TRAE: treatment-related adverse events; TRD: treatment-related deaths.

## Supplementary Materials

The following supporting information can be downloaded at: https://www.mdpi.com/article/10.3390/cancers14020441/s1, Table S1: Overview of unspecific immunomodulating therapies. Figure S1: Risk of bias assessment of included randomized controlled trials—individual trial judgements. Figure S2: Risk of bias assessment of included randomized controlled trials—collective risk of bias assessments. Figure S3: Risk of bias assessment of included single-arm cohort trials—individual trial judgements. Figure S4: Risk of bias assessment of included single-arm cohort trials—collective risk of bias assessments. Appendix A: Complete search strategies for Ovid, Web of Science and the Cochrane Library.

## References

[1] Sung H., Ferlay J., Siegel R.L., Laversanne M., Soerjomataram I., Jemal A., Bray F. (2021). Global Cancer Statistics 2020: GLOBOCAN Estimates of Incidence and Mortality Worldwide for 36 Cancers in 185 Countries. CA Cancer J. Clin..

[2] Leitlinienprogramm Onkologie (Deutsche Krebsgesellschaft, D.K.; AWMF) (2021). S3-Leitline Diagnostik, Therapie und Nachsorge der Patientin mit Zervixkarzinom, Langversion 2.0. https://www.awmf.org/uploads/tx_szleitlinien/032-033OLl_S3_Diagnostik_Therapie_Nachsorge_Zervixkarzinom_2021-05.pdf.

[3] Cohen E.E.W., Moore K.N., Slomovitz B.M., Chung C.H., Anderson M.L., Morris S.R., Mauro D., Burtness B. (2015). Phase I/II study of ADXS11-001 or MEDI4736 immunotherapies alone and in combination, in patients with recurrent/metastatic cervical or human papillomavirus (HPV)-positive head and neck cancer. J. Immunother. Cancer.

[4] Tewari K.S., Sill M.W., Penson R.T., Huang H., Ramondetta L.M., Landrum L.M., Oaknin A., Reid T.J., Leitao M.M., Michael H.E. (2017). Bevacizumab for advanced cervical cancer: Final overall survival and adverse event analysis of a randomised, controlled, open-label, phase 3 trial (Gynecologic Oncology Group 240). Lancet.

[5] Monk B.J., Sill M.W., Burger R.A., Gray H.J., Buekers T.E., Roman L.D. (2009). Phase II trial of bevacizumab in the treatment of persistent or recurrent squamous cell carcinoma of the cervix: A gynecologic oncology group study. J. Clin. Oncol..

[6] Alberts D.S., Blessing J.A., Landrum L.M., Warshal D.P., Martin L.P., Rose S.L., Bonebrake A.J., Ramondetta L.M. (2012). Phase II trial of nab-paclitaxel in the treatment of recurrent or persistent advanced cervix cancer: A gynecologic oncology group study. Gynecol. Oncol..

[7] Santin A.D., Sill M.W., McMeekin D.S., Leitao M.M., Brown J., Sutton G.P., Van Le L., Griffin P., Boardman C.H. (2011). Phase II trial of cetuximab in the treatment of persistent or recurrent squamous or non-squamous cell carcinoma of the cervix: A Gynecologic Oncology Group study. Gynecol. Oncol..

[8] National Institute of Cancer Immunotherapy. https://www.cancer.gov/publications/dictionaries/cancer-terms/def/immunotherapy.

[9] Schepisi G., Cadadei C., Toma I., Poti G., Iaia M.L., Farolfi A., Conteduca V., Lolli C., Ravaglia G., Brighi N. (2021). Immunotherapy and its development for gynecological (ovarian, endometrial and cervical) tumors: From immune checkpoint inhibitors to Chimeric Antigen Receptor (CAR)-T cell therapy. Cancers.

[10] Moher D., Liberati A., Tetzlaff J., Altman D.G., The PRISMA Group (2009). Preferred reporting items for systematic reviews and meta-analyses: The PRISMA statement. PLoS Med..

[11] Goossen K., Tenckhoff S., Probst P., Grummich K., Mihaljevic A.L., Buechler M.W., Diener M.K. (2018). Optimal literature search for systematic reviews in surgery. Langenbeck’s Arch. Surg..

[12] Santos C.M.d.C., Pimenta C.A.d.M., Nobre M.R.C. (2007). The PICO strategy for the research question construction and evidence search. Rev. Lat.-Am. Enferm..

[13] Sterne J.A., Savovic J., Page M., Elbers R., Boutron I., Cates C., Cheng V., Corbett M., Eldrige S., Emberson J. (2019). RoB 2: A revised tool for assessing risk of bias in randomised trials. BMJ.

[14] Sterne J.A., Hernan M.A., Reeves B.C., Savovic J., Berkman N.D., Viswanathan M., Henry D., Altman D.G., Ansari M.T., Boutron A. (2016). ROBINS-I: A tool for assessing risk of bias in non-randomised studies of interventions. BMJ.

[15] Jullien S., Ryan H., Modi M., Bhatia R. (2016). Six months therapy for tuberculous meningitis. Cochrane Database Syst. Rev..

[16] Marin-Acevedo J.A., Dholaria B., Soyano A.E., Knutson K.L., Chumsri S., Lou Y. (2018). Next generation of immune checkpoint therapy in cancer: New developments and challenges. J. Hematol. Oncol..

[17] Colombo N., Dubot C., Lorusso D., Caceres M.V., Hasegawa K., Shapira-Frommer R., Tewari K.S., Salman P., Hoyos Usta E., Yanez E. (2021). Pembrolizumab for Persistent, Recurrent, or Metastatic Cervical Cancer. N. Engl. J. Med..

[18] Chung H.C., Ros W., Delord J.-P., Perets R., Italiano A., Shapira-Frommer R., Manzuk L., Piha-Paul S.A., Xu L., Zeigenfuss S. (2019). Efficacy and Safety of Pembrolizumab in Previously Treated Advanced Cervical Cancer: Results From the Phase II KEYNOTE-158 Study. J. Clin. Oncol. Off. J. Am. Soc. Clin. Oncol..

[19] Frenel J.-S., Le Tourneau C., O’Neil B., Ott P.A., Piha-Paul S.A., Gomez-Roca C., Van Brummelen M.J., Rugo H.S., Thomas S., Saraf S. (2017). Safety and Efficacy of Pembrolizumab in Advanced, Programmed Death Ligand 1-Positive Cervical Cancer: Results From the Phase Ib KEYNOTE-028 Trial. J. Clin. Oncol. Off. J. Am. Soc. Clin. Oncol..

[20] Duska L.R., Scalici J.M., Temkin S.M., Schwarz J.K., Crane E.K., Moxley K.M., Hamiltion C.A., Wethington S.L., Petronie G.R., Varhegyi N.E. (2020). Results of an early safety analysis of a study of the combination of pembrolizumab and pelvic chemoradiation in locally advanced cervical cancer. Cancer.

[21] Youn J.W., Hur S.-Y., Woo J.W., Kim Y.-M., Lim M.C., Park S.Y., Seo S.S., No J.H., Kim B.-G., Lee J.-K. (2020). Pembrolizumab plus GX-188E therapeutic DNA vaccine in patients with HPV-16-positive or HPV-18-positive advanced cervical cancer: Interim results of a single-arm, phase 2 trial. Lancet Oncol..

[22] Frumovitz M., Westin S.N., Salvo G., Zarifa A., Xu M., Yap T.A., Rodon A.J., Karp D.D., Abonofal A., Jazaeri A. (2020). Phase II study of pembrolizumab efficacy and safety in women with recurrent small cell neuroendocrine carcinoma of the lower genital tract. Gynecol. Oncol..

[23] Qiao G., Wang X., Zhou X., Morse M.A., Wu J., Wang S., Song Y., Jang N., Zhao Y., Zhou L. (2019). Immune correlates of clinical benefit in a phase I study of hyperthermia with adoptive T cell immunotherapy in patients with solid tumors. Int. J. Hyperth..

[24] Santin A.D., Deng W., Frumovitz M., Buza N., Bellone S., Huh W., Khleif S., Lankes H.A., Ratner E.S., O’Cearbhaill R.E. (2020). Phase II evaluation of nivolumab in the treatment of persistent or recurrent cervical cancer (NCT02257528/NRG-GY002). Gynecol. Oncol..

[25] Tamura K., Hasegawa K., Katsumata N., Matsumoto K., Mukai H., Takahashi S., Nomura H., Minami H. (2019). Efficacy and safety of nivolumab in Japanese patients with uterine cervical cancer, uterine corpus cancer, or soft tissue sarcoma: Multicenter, open-label phase 2 trial. Cancer Sci..

[26] Naumann R.W., Hollebecque A., Meyer T., Devlin M.-J., Oaknin A., Kerger J., Lopez-Picazo J.M., Machiels J.-P., Delord J.-P., Evans T.R.J. (2019). Safety and Efficacy of Nivolumab Monotherapy in Recurrent or Metastatic Cervical, Vaginal, or Vulvar Carcinoma: Results From the Phase I/II CheckMate 358 Trial. J. Clin. Oncol. Off. J. Am. Soc. Clin. Oncol..

[27] Yin H., Guo W., Sun X., Li R., Feng C., Tan Y. (2020). TILs and Anti-PD1 Therapy: An Alternative Combination Therapy for PDL1 Negative Metastatic Cervical Cancer. J. Immunol. Res..

[28] Friedman C.F., Snyder Charen A., Zhou Q., Carducci M.A., Buckley De Meritens A., Corr B.R., Fu S., Hollmann T.J., Iasonos A., Konner J.A. (2020). Phase II study of atezolizumab in combination with bevacizumab in patients with advanced cervical cancer. J. Immunother. Cancer.

[29] Jung K.H., LoRusso P., Burris H., Gordon M., Bang Y.-J., Hellmann M.D., Cervantes A., Ochoa de Olza M., Marabelle A., Hodi F.S. (2019). Phase I Study of the Indoleamine 2,3-Dioxygenase 1 (IDO1) Inhibitor Navoximod (GDC-0919) Administered with PD-L1 Inhibitor (Atezolizumab) in Advanced Solid Tumors. Clin. Cancer Res. Off. J. Am. Assoc. Cancer Res..

[30] Mayadev J.S., Enserro D., Lin Y.G., Da Silva D.M., Lankes H.A., Aghajanian C., Ghamande S., Moore K.N., Kennedy V.A., Fracasso P.M. (2020). Sequential Ipilimumab After Chemoradiotherapy in Curative-Intent Treatment of Patients With Node-Positive Cervical Cancer. Jama Oncol..

[31] Lheureux S., Butler M.O., Clarke B., Cristea M.C., Martin L.P., Tonkin K., Fleming G.F., Tinker A.V., Hirte H.W., Tsoref D. (2018). Association of Ipilimumab With Safety and Antitumor Activity in Women With Metastatic or Recurrent Human Papillomavirus-Related Cervical Carcinoma. JAMA Oncol..

[32] Lan C., Shen J., Wang Y., Li J., Liu Z., He M., Cao X., Ling J., Huang J., Zheng M. (2020). Camrelizumab Plus Apatinib in Patients With Advanced Cervical Cancer (CLAP): A Multicenter, Open-Label, Single-Arm, Phase II Trial. J. Clin. Oncol. Off. J. Am. Soc. Clin. Oncol..

[33] Rischin D., Gil-Martin M., Gonzalez-Martin A., Brana I., Hou J.Y., Cho D., Falchook G.S., Formenti S., Jabbour S., Moore K. (2020). PD-1 blockade in recurrent or metastatic cervical cancer: Data from cemiplimab phase I expansion cohorts and characterization of PD-L1 expression in cervical cancer. Gynecol. Oncol..

[34] O’Malley D.M., Oaknin A., Monk B.J., Selle F., Rojas C., Gladieff L., Berton D., Leary A., Moore K.N., Estevez-Diz M.D.P. (2021). Phase II study of the safety and efficacy of the anti-PD-1 antibody balstilimab in patients with recurrent and/or metastatic cervical cancer. Gynecol. Oncol..

[35] Strauss J., Gatti-Mays M.E., Chul Cho B., Hill A., Salas S., McClay E., Redman J.M., Sater H.A., Donahue R.N., Jochems C. (2020). Bintrafusp alfa, a bifunctional fusion protein targeting TGF-beta and PD-L1, in patients with human papillomavirus-associated malignancies. J. Immunother. Cancer.

[36] Tinker A.V., Hirte H.W., Provencher D., Butler M., Ritter H., Tu D., Azim H.A., Paralejas P., Grenier N., Hahn S.-A. (2019). Dose-Ranging and Cohort-Expansion Study of Monalizumab (IPH2201) in Patients with Advanced Gynecologic Malignancies: A Trial of the Canadian Cancer Trials Group (CCTG): IND221. Clin. Cancer Res. Off. J. Am. Assoc. Cancer Res..

[37] Chung H., Delord J.-P., Perets R., Italiano A., Shapira-Frommer R., Manzuk L., Piha-Paul S., Xu L., Jin F., Norwood K. (2021). Pembrolizumab treatment of advanced cervical cancer: Updated results from the phase II KEYNOTE-158 study. Gynecol. Oncol..

[38] Luostarinen T., Apter D., Dillner J., Eriksson T., Harjula K., Natunen K., Paavonen J., Pukkala E., Lehtinen M. (2018). Vaccination protects against invasive HPV-associated cancers. Int. J. Cancer.

[39] Hu Z., Ott P.A., Wu C.J. (2018). Towards personalized, tumour-specific, therapeutic vaccines for cancer. Nat. Rev. Immunol..

[40] Narisawa-Saito M., Kiyono T. (2007). Basic mechanisms of high-risk human papillomavirus-induced carcinogenesis: Roles of E6 and E7 proteins. Cancer Sci..

[41] Hasegawa K., Ikeda Y., Kunugi Y., Kurosaki A., Imai Y., Kohyama S., Nagao S., Kozawa E., Yoshida K., Tsunoda T. (2018). Phase I Study of Multiple Epitope Peptide Vaccination in Patients With Recurrent or Persistent Cervical Cancer. J. Immunother..

[42] Kenter G.G., Welters M.J.P., Valentijn A.R.P.M., Löwik M.J.G., Berends-van der Meer D.M.A., Vloon A.P.G., Drijfhout J.W., Wafelman A.R., Oostendorp J., Fleuren G.J. (2008). Phase I immunotherapeutic trial with long peptides spanning the E6 and E7 sequences of high-risk human papillomavirus 16 in end-stage cervical cancer patients shows low toxicity and robust immunogenicity. Clin. Cancer Res. Off. J. Am. Assoc. Cancer Res..

[43] Melief C.J.M., Welters M.J.P., Vergote I., Kroep J.R., Kenter G.G., Ottevanger P.B., Tjalma W.A.A., Denys H., van Poelgeest M.I.E., Nijman H.W. (2020). Strong vaccine responses during chemotherapy are associated with prolonged cancer survival. Sci. Transl. Med..

[44] Reuschenbach M., Pauligk C., Karbach J., Rafiyan M.-R., Kloor M., Prigge E.-S., Sauer M., Al-Batran S.-E., Kaufmann A.M., Schneider A. (2016). A phase 1/2a study to test the safety and immunogenicity of a p16(INK4a) peptide vaccine in patients with advanced human papillomavirus-associated cancers. Cancer.

[45] Steller M.A., Gurski K.J., Murakami M., Daniel R.W., Shah K.V., Celis E., Sette A., Trimble E.L., Park R.C., Marincola F.M. (1998). Cell-mediated immunological responses in cervical and vaginal cancer patients immunized with a lipidated epitope of human papillomavirus type 16 E7. Clin. Cancer Res. Off. J. Am. Assoc. Cancer Res..

[46] Takeuchi S., Kagabu M., Shoji T., Nitta Y., Sugiyama T., Sato J., Nakamura Y. (2020). Anti-cancer immunotherapy using cancer-derived multiple epitope-peptides cocktail vaccination clinical studies in patients with refractory/persistent disease of uterine cervical cancer and ovarian cancer phase 2. Oncoimmunology.

[47] Tsuda N., Mochizuki K., Harada M., Sukehiro A., Kawano K., Yamada A., Ushijima K., Sugiyama T., Nishida T., Yamana H. (2004). Vaccination with predesignated or evidence-based peptides for patients with recurrent gynecologic cancers. J. Immunother..

[48] van Driel W.J., Ressing M.E., Kenter G.G., Brandt R.M., Krul E.J., van Rossum A.B., Schuuring E., Offringa R., Bauknecht T., Tamm-Hermelink A. (1999). Vaccination with HPV16 peptides of patients with advanced cervical carcinoma: Clinical evaluation of a phase I-II trial. Eur. J. Cancer.

[49] van Poelgeest M.I.E., Welters M.J.P., van Esch E.M.G., Stynenbosch L.F.M., Kerpershoek G., van Persijn van Meerten E.L., van den Hende M., Lowik M.J.G., Berends-van der Meer D.M.A., Fathers L.M. (2013). HPV16 synthetic long peptide (HPV16-SLP) vaccination therapy of patients with advanced or recurrent HPV16-induced gynecological carcinoma, a phase II trial. J. Transl. Med..

[50] Welters M.J.P., Kenter G.G., Piersma S.J., Vloon A.P.G., Lowik M.J.G., Berends-van der Meer D.M.A., Drijfhout J.W., Valentijn A.R.P.M., Wafelman A.R., Oostendorp J. (2008). Induction of tumor-specific CD4+ and CD8+ T-cell immunity in cervical cancer patients by a human papillomavirus type 16 E6 and E7 long peptides vaccine. Clin. Cancer Res. Off. J. Am. Assoc. Cancer Res..

[51] Welters M.J., van der Sluis T.C., van Meir H., Loof N.M., van Ham V.J., van Duikeren S., Santegoets S.J., Arens R., de Kam M.L., Cohen A.F. (2016). Vaccination during myeloid cell depletion by cancer chemotherapy fosters robust T cell responses. Sci. Transl. Med..

[52] Choi C.H., Choi H.J., Lee J.-W., Kang E.-S., Cho D., Park B.K., Kim Y.-M., Kim D.-Y., Seo H., Park M. (2020). Phase I Study of a B Cell-Based and Monocyte-Based Immunotherapeutic Vaccine, BVAC-C in Human Papillomavirus Type 16- or 18-Positive Recurrent Cervical Cancer. J. Clin. Med..

[53] Ferrara A., Nonn M., Sehr P., Schreckenberger C., Pawlita M., Durst M., Schneider A., Kaufmann A.M. (2003). Dendritic cell-based tumor vaccine for cervical cancer II: Results of a clinical pilot study in 15 individual patients. J. Cancer Res. Clin. Oncol..

[54] Rahma O.E., Herrin V.E., Ibrahim R.A., Toubaji A., Bernstein S., Dakheel O., Steinberg S.M., Abu Eid R., Mkrtichyan M., Berzofsky J.A. (2014). Pre-immature dendritic cells (PIDC) pulsed with HPV16 E6 or E7 peptide are capable of eliciting specific immune response in patients with advanced cervical cancer. J. Transl. Med..

[55] Ramanathan P., Ganeshrajah S., Raghanvan R.K., Singh S.S., Thangarajan R. (2014). Development and Clinical Evaluation of Dendritic Cell Vaccines for HPV Related Cervical Cancer—A Feasibility Study. Asian Pac. J. Cancer Prev..

[56] Santin A.D., Bellone S., Palmieri M., Ravaggi A., Romani C., Tassi R., Roman J.J., Burnett A., Pecorelli S., Cannon M.J. (2006). HPV16/18 E7-pulsed dendritic cell vaccination in cervical cancer patients with recurrent disease refractory to standard treatment modalities. Gynecol. Oncol..

[57] Santin A.D., Bellone S., Palmieri M., Zanolini A., Ravaggi A., Siegel E.R., Roman J.J., Pecorelli S., Cannon M.J. (2008). Human papillomavirus type 16 and 18 E7-pulsed dendritic cell vaccination of stage IB or IIA cervical cancer patients: A phase I escalating-dose trial. J. Virol..

[58] Hasan Y., Furtado L., Tergas A., Lee N., Brooks R., McCall A., Golden D., Jolly S., Fleming G., Morrow M. (2020). A Phase 1 Trial Assessing the Safety and Tolerability of a Therapeutic DNA Vaccination Against HPV16 and HPV18 E6/E7 Oncogenes After Chemoradiation for Cervical Cancer. Int. J. Radiat. Oncol. Biol. Phys..

[59] Hui K.M., Ang P.T., Huang L., Tay S.K. (1997). Phase I study of immunotherapy of cutaneous metastases of human carcinoma using allogeneic and xenogeneic MHC DNA-liposome complexes. Gene Ther..

[60] Borysiewicz L.K., Fiander A., Nimako M., Man S., Wilkinson G.W., Westmoreland D., Evans A.S., Adams M., Stacey S.N., Boursnell M.E. (1996). A recombinant vaccinia virus encoding human papillomavirus types 16 and 18, E6 and E7 proteins as immunotherapy for cervical cancer. Lancet.

[61] Freedman R.S., Bowen J.M., Atkinson E.N., Wallace S., Lotzová E., Silva E., Edwards C.L., Delclos L., Scott W., Patenia B. (1989). Randomized comparison of viral oncolysate plus radiation and radiation alone in uterine cervix carcinoma. Am. J. Clin. Oncol..

[62] Basu P., Mehta A., Jain M., Gupta S., Nagarkar R.V., John S., Petit R. (2018). A Randomized Phase 2 Study of ADXS11-001 Listeria monocytogenes-Listeriolysin O Immunotherapy With or Without Cisplatin in Treatment of Advanced Cervical Cancer. Int. J. Gynecol. Cancer.

[63] Huh W.K., Brady W.E., Fracasso P.M., Dizon D.S., Powell M.A., Monk B.J., Leath C.A., Landrum L.M., Tanner E.J., Crane E.K. (2020). Phase II study of axalimogene filolisbac (ADXS-HPV) for platinum-refractory cervical carcinoma: An NRG oncology/gynecologic oncology group study. Gynecol. Oncol..

[64] Maciag P.C., Radulovic S., Rothman J. (2009). The first clinical use of a live-attenuated Listeria monocytogenes vaccine: A Phase I safety study of Lm-LLO-E7 in patients with advanced carcinoma of the cervix. Vaccine.

[65] Waldman A.D., Fritz J.M., Lenardo M.J. (2020). A guide to cancer immunotherapy: From T cell basic science to clinical practice. Nat. Rev. Immunol..

[66] Garrido F., Aptsiauri N., Doorduijn E.M., Lora A.M.G., van Hall T. (2016). The urgent need to recover MHC class I in cancers for effective immunotherapy. Curr. Opin. Immunol..

[67] Chen B., Liu L., Xu H., Yang Y., Zhang L., Zhang F. (2015). Effectiveness of immune therapy combined with chemotherapy on the immune function and recurrence rate of cervical cancer. Exp. Ther. Med..

[68] Li N., Tian Y.-W., Xu Y., Meng D.-D., Gao L., Shen W.-J., Liu Z.-L., Xu Z.-Q. (2019). Combined Treatment with Autologous CIK Cells, Radiotherapy and Chemotherapy in Advanced Cervical Cancer. Pathol. Oncol. Res. POR.

[69] Stevanovic S., Draper L.M., Langhan M.M., Campbell T.E., Kwong M.L., Wunderlich J.R., Dudley M.E., Yang J.C., Sherry R.M., Kammula U.S. (2015). Complete Regression of Metastatic Cervical Cancer After Treatment With Human Papillomavirus-Targeted Tumor-Infiltrating T Cells. J. Clin. Oncol..

[70] Stevanovic S., Helman S.R., Wunderlich J.R., Langhan M.M., Doran S.L., Kwong M.L.M., Somerville R.P.T., Klebanoff C.A., Kammula U.S., Sherry R.M. (2019). A Phase II Study of Tumor-infiltrating Lymphocyte Therapy for Human Papillomavirus-associated Epithelial Cancers. Clin. Cancer Res. Off. J. Am. Assoc. Cancer Res..

[71] Doran S.L., Stevanovic S., Adhikary S., Gartner J.J., Jia L., Kwong M.L.M., Faquin W.C., Hewitt S.M., Sherry R.M., Yang J.C. (2019). T-Cell Receptor Gene Therapy for Human Papillomavirus-Associated Epithelial Cancers: A First-in-Human, Phase I/II Study. J. Clin. Oncol..

[72] Lu Y.-C., Parker L.L., Lu T., Zheng Z., Toomey M.A., White D.E., Yao X., Li Y.F., Robbins P.F., Feldman S.A. (2017). Treatment of Patients With Metastatic Cancer Using a Major Histocompatibility Complex Class II-Restricted T-Cell Receptor Targeting the Cancer Germline Antigen MAGE-A3. J. Clin. Oncol. Off. J. Am. Soc. Clin. Oncol..

[73] Nagarsheth N.B., Norberg S.M., Sinkoe A.L., Adhikary S., Meyer T.J., Lack J.B., Warner A.C., Schweitzer C., Doran S.L., Korrapati S. (2021). TCR-engineered T cells targeting E7 for patients with metastatic HPV-associated epithelial cancers. Nat. Med..

[74] Wadler S., Burk R.D., Neuberg D., Rameau R., Runowicz C.D., Goldberg G., McGill F., Tachezy R., Comis R., Edmonson J. (1995). Lack of efficacy of interferon-α therapy in recurrent, advanced cervical cancer. J. Interferon Cytokine Res..

[75] Wadler S., Schwartz E.L., Haynes H., Rameau R., Quish A., Mandeli J., Gallagher R., Hallam S., Fields A., Goldberg G. (1997). All-trans retinoic acid and interferon-α-2a in patients with metastatic or recurrent carcinoma of the uterine cervix: Clinical and pharmacokinetic studies. Cancer.

[76] Wilailak S., Dangprasert S., Srisupundit S. (2003). Phase I clinical trial of chemoimmunotherapy in combination with radiotherapy in stage IIIB cervical cancer patients. Int. J. Gynecol. Cancer Off. J. Int. Gynecol. Cancer Soc..

[77] Look K.Y., Blessing J.A., Nelson B.E., Johnson G.A., Fowler W.C.J., Reid G.C. (1998). A Phase II Trial of Isotretinoin and Alpha Interferon in Patients With Recurrent Squamous Cell Carcinoma of the Cervix: A Gynecologic Oncology Group Study. Am. J. Clin. Oncol..

[78] Braud A.-C., Gonzague L., Bertucci F., Genre D., Camerlo J., Gravis G., Goncalves A., Moutardier V., Viret F., Maraninchi D. (2002). Retinoids, cisplatin and interferon-alpha in recurrent or metastatic cervical squamous cell carcinoma: Clinical results of 2 phase II trials. Eur. Cytokine Netw..

[79] Lippman S.M., Kavanagh J.J., Paredes-Espinoza M., Delgadillo-Madrueño F., Paredes-Casillas P., Hong W.K., Holderner E., Krakoff I.H. (1992). 13-cis-Retinoic Acid Plus Interferon α-2a: Highly Active Systemic Theraphy for Squamous Cell Carcinoma of the Cervix. JNCI J. Natl. Cancer Inst..

[80] Duenas-Gonzalez A., Verastegui E., Lopez-Graniel C., Gonzalez A., Mota A., Barrera-Franco J.L., Meneses A., Chanona J., de la Garza J., Chavez-Blanco A. (2002). A pilot study of perilymphatic leukocyte cytokine mixture (IRX-2) as neoadjuvant treatment for early stage cervical carcinoma. Int. Immunopharmacol..

[81] Wadler S., Levy D., Frederickson H.L., Falkson C.I., Wang Y., Weller E., Burk R., Ho G., Kadish A.S. (2004). A phase II trial of interleukin-12 in patients with advanced cervical cancer: Clinical and immunologic correlates. Eastern Cooperative Oncology Group study E1E96. Gynecol. Oncol..

[82] Cappello F., Corradi, Meli G. (1984). Use of B.C.G. as loco-regional aspecific immunostimulator in cervical carcinoma. Clin. Exp. Obstet. Gynecol..

[83] DiSaia P.J., Bundy B.N., Curry S.L., Schlaerth J., Thigpen J.T. (1987). Phase III study on the treatment of women with cervical cancer, stage IIB, IIIB, and IVA (confined to the pelvis and/or periaortic nodes), with radiotherapy alone versus radiotherapy plus immunotherapy with intravenous Corynebacterium parvum: A Gynecologic Oncology Group Study. Gynecol. Oncol..

[84] Mignot M.H., Lens J.W., Drexhage H.A., von Blomberg B.M., Flier V.D., Oort J., Stolk J.G. (1981). Lower relapse rates after neighbourhood injection of Corynebacterium parvum in operable cervix carcinoma. Br. J. Cancer.

[85] Gall S.A., DiSaia P.J., Schmidt H., Mittelstaedt L., Newman P., Creasman W. (1978). Toxicity manifestations following intravenous Corynebacterium parvum administration to patients with ovarian and cervical carcinoma. Am. J. Obstet. Gynecol..

[86] Ahn W.S., Kim D.J., Chae G.T., Lee J.M., Bae S.M., Sin J.I., Kim Y.W., Namkoong S.E., Lee I.P. (2004). Natural killer cell activity and quality of life were improved by consumption of a mushroom extract, Agaricus blazei Murill Kyowa, in gynecological cancer patients undergoing chemotherapy. Int. J. Gynecol. Cancer.

[87] Kikkawa F., Kawai M., Oguchi H., Kojima M., Ishikawa H., Iwata M., Maeda O., Tomoda Y., Arii Y., Kuzuya K. (1993). Randomised study of immunotherapy with OK-432 in uterine cervical carcinoma. Eur. J. Cancer.

[88] Noda K., Teshima K., Tekeuti K., Hasegawa K., Inoue K., Yamashita K., Sawaragi I., Nakajima T., Takashima E., Ikeuchi M. (1989). Immunotherapy using the streptococcal preparation OK-432 for the treatment of uterine cervical cancer. Cervical Cancer Immunotherapy Study Group. Gynecol. Oncol..

[89] Okamura K., Hamazaki Y., Yajima A., Noda K. (1989). Adjuvant immunotherapy: Two randomized controlled studies of patients with cervical cancer. Biomed. Pharmacother..

[90] Kucera H., Micksche M. (1982). Adjuvanticity of vitamin A in advanced irradiated cervical cancer. Fortschr. Der Med..

[91] Mallmann P., Krebs D. (1989). The effect of immunotherapy with thymopentin on the parameters of cellular immunity and the clinical course of gynecologic tumor patients. Onkologie.

[92] Noda K., Takeuchi S., Yajima A., Akiya K., Kasamatsu T., Tomoda Y., Ozawa M., Sekiba K., Sugimori H., Hashimoto S. (1992). Clinical effect of sizofiran combined with irradiation in cervical cancer patients: A randomized controlled study. Cooperative Study Group on SPG for Gynecological Cancer. Jpn. J. Clin. Oncol..

[93] Noda K., Ohashi Y., Okada H., Ogita S., Ozaki M., Kikuchi Y., Takegawa Y., Niibe H., Fujii S., Horiuchi J. (2006). Randomized phase II study of immunomodulator Z-100 in patients with stage IIIB cervical cancer with radiation therapy. Jpn. J. Clin. Oncol..

[94] Noda K., Ohashi Y., Sugimori H., Ozaki M., Niibe H., Ogita S., Kohno I., Hasegawa K., Kikuchi Y., Takegawa Y. (2006). Phase III double-blind randomized trial of radiation therapy for stage IIIb cervical cancer in combination with low-or high-dose Z-100: Treatment with immunomodulator, more is not better. Gynecol. Oncol..

[95] Sugiyama T., Fujiwara K., Ohashi Y., Yokota H., Hatae M., Ohno T., Nagai Y., Mitsuhashi N., Ochiai K., Noda K. (2014). Phase III placebo-controlled double-blind randomized trial of radiotherapy for stage IIB-IVA cervical cancer with or without immunomodulator Z-100: A JGOG study. Ann. Oncol..

[96] Okawa T., Kita M., Arai T., Iida K., Dokiya T., Takegawa Y., Hirokawa Y., Yamazaki K., Hashimoto S. (1989). Phase II randomized clinical trial of LC9018 concurrently used with radiation in the treatment of carcinoma of the uterine cervix. Its effect on tumor reduction and histology. Cancer.

[97] Okawa T., Niibe H., Arai T., Sekiba K., Noda K., Takeuchi S., Hashimoto S., Ogawa N. (1993). Effect of LC9018 combined with radiation therapy on carcinoma of the uterine cervix. A phase III, multicenter, randomized, controlled study. Cancer.

[98] Tewari K.S., Monk B.J., Vergote I., Miller A., de Melo A.C., Kim H.S., Kim Y.M., Lisyanskaya A., Samouëlian V., Lorusso D. (2021). VP4-2021: EMPOWER-Cervical 1/GOG-3016/ENGOT-cx9: Interim analysis of phase III trial of cemiplimab vs. investigator’s choice (IC) chemotherapy (chemo) in recurrent/metastatic (R/M) cervical carcinoma. Ann. Oncol..

[99] Otter S., Chatterjee J., Stewart A., Michael A. (2019). The role of biomarkers for the prediction of response to checkpoint immunotherapy and the rationale for the use of checkpoint immunotherapy in cervical cancer. Clin. Oncol..

[100] Naumann R.W., Oaknin A., Meyer T., Lopez-Picazo J.M., Lao C., Bang Y.J., Boni V., Sharfman W.H., Park J.C., Devriese L.A. (2019). Efficacy and safety of nivolumab (Nivo) + ipilimumab (Ipi) in patients (pts) with recurrent/metastatic (R/M) cervical cancer: Results from CheckMate 358. Ann. Oncol..

[101] Tseng Y.-J., Lee C.H., Chen W.Y., Yang J.L., Tzeng H.T. (2021). Inhibition of PAI-1 Blocks PD-L1 Endocytosis and Improves the Response of Melanoma Cells to Immune Checkpoint Blockade. J. Investig. Dermatol..

[102] Martins F., Sofiya L., Sykiotis G.P., Lamine F., Maillard M., Fraga M., Shabafrouz K., Ribi C., Cairoli A., Guex-Crosier Y. (2019). Adverse effects of immune-checkpoint inhibitors: Epidemiology, management and surveillance. Nat. Rev. Clin. Oncol..

[103] Rumfield C.S., Roller N., Pellom S.T., Schlom J., Jochems C. (2020). Therapeutic vaccines for HPV-associated malignancies. ImmunoTargets Ther..

[104] Coleman S., Clayton A., Mason M.D., Jasani B., Adams M., Tabi Z. (2005). Recovery of CD8+ T-cell function during systemic chemotherapy in advanced ovarian cancer. Cancer Res..

[105] Wu X., Feng Q.-M., Wang Y., Shi J., Ge H.-L., Di W. (2010). The immunologic aspects in advanced ovarian cancer patients treated with paclitaxel and carboplatin chemotherapy. Cancer Immunol. Immunother..

[106] Lorusso D., Petrelli F., Coinu A., Raspagliesi F., Barni S. (2014). A systematic review comparing cisplatin and carboplatin plus paclitaxel-based chemotherapy for recurrent or metastatic cervical cancer. Gynecol. Oncol..

[107] Strauss J., Floudas C.S., Sater H.A., Manu M., Lamping E., Francis D.C., Cordes L.M., Marte J., Donahue R.N., Jochems C. (2021). Phase II evaluation of the triple combination of PDS0101, M9241, and bintrafusp alfa in patients with HPV 16 positive malignancies. J. Clin. Oncol..

[108] Boilesen D.R., Nielsen K.N., Holst P.J. (2021). Novel Antigenic Targets of HPV Therapeutic Vaccines. Vaccines.

[109] Wang Z.-X., Cao J.-X., Wang M., Li D., Cui Y.-X., Zhang X.-Y., Liu J.-L., Li J.-L. (2014). Adoptive cellular immunotherapy for the treatment of patients with breast cancer: A meta-analysis. Cytotherapy.

[110] Dafni U., Michielin O., Lluesma S.M., Tsourti Z., Polydoropoulou V., Karlis D., Besser M., Haanen J., Svane I.-M., Ohashi P. (2019). Efficacy of adoptive therapy with tumor-infiltrating lymphocytes and recombinant interleukin-2 in advanced cutaneous melanoma: A systematic review and meta-analysis. Ann. Oncol..

[111] Sutton G.P., Blessing J.A., McGuire W.P., Patton T., Look K.Y. (1993). Phase II trial of ifosfamide and mesna in patients with advanced or recurrent squamous carcinoma of the cervix who had never received chemotherapy: A Gynecologic Oncology Group study. Am. J. Obstet. Gynecol..

[112] Sutton G.P., Blessing J.A., Photopulos G., Berman M.L., Homesley H.D. (1990). Early phase II Gynecologic Oncology Group experience with ifosfamide/mesna in gynecologic malignancies. Cancer Chemother. Pharmacol..

[113] Cao J., Chen C., Wang Y., Chen X., Chen Z., Luo X. (2016). Influence of autologous dendritic cells on cytokine-induced killer cell proliferation, cell phenotype and antitumor activity in vitro. Oncol. Lett..

[114] Zheng C., Yu G., Wang H., Tang A., Geng P., Zhang H., Zhu Z., Li F., Xie X. (2015). Meta-analysis of chemotherapy and dendritic cells with cytokine-induced killer cells in the treatment of non-small-cell lung cancer. Int. J. Clin. Exp. Med..

[115] Mu Y., Zhou C.-H., Chen S.-F., Ding J., Zhang Y.-X., Yang Y.-P., Wang W.-H. (2016). Effectiveness and safety of chemotherapy combined with cytokine-induced killer cell/dendritic cell–cytokine-induced killer cell therapy for treatment of gastric cancer in China: A systematic review and meta-analysis. Cytotherapy.

[116] Wang Z.-X., Cao J.-X., Liu Z.-P., Cui Y.-X., Li C.-Y., Li D., Zhang X.-Y., Liu J.-L., Li J.-L. (2014). Combination of chemotherapy and immunotherapy for colon cancer in China: A meta-analysis. World J. Gastroenterol. WJG.

