# Supplementary material for: Efficacy and Safety of Immunotherapy for Cervical Cancer—A Systematic Review of Clinical Trials
Source: Cancers (Basel). 2022 Jan 17;14(2):441. doi: 10.3390/cancers14020441 (PMC8773848; doi:10.3390/cancers14020441)
Supplement: Supplementary file 1 [file cancers-14-00441-s001.zip › cancers-1544474-supplementary.pdf]

cancers

Systematic Review

Efficacy and Safety of Immunotherapy for Cervical Cancer—A Systematic Review of Clinical Trials

Mona W. Schmidt [1,2,*], Marco J. Battista [1], Marcus Schmidt [1], Monique Garcia [2,3], Timo Siepmann [2,4], Annette Hasenburg [1] and Katharina Anic [1]

1 Department of Gynecology and Obstetrics, University Medical Centre Mainz, Langenbeckstraße 1, 55131 Mainz, Germany; marco.battista@unimedizin-mainz.de (M.J.B.); marcus.schmidt@unimedizin-mainz.de (M.S.); annette.hasenburg@unimedizin-mainz.de (A.H.); katharina.anic@unimedizin-mainz.de (K.A.)
2 Division of Health Care Sciences Center for Clinical Research and Management Education Dresden, Dresden International University, 01067 Dresden, Germany; med.moniquegarcia@gmail.com (M.G.); timo.siepmann@uniklinikum-dresden.de (T.S.)
3 Department of Medicine, Pontifícia Universidade Católica de Minas Gerais (PUC MG), 32604-115 Betim, Brazil
4 Department of Neurology, University Hospital Carl Gustav Carus, Technische Universität Dresden, 01307 Dresden, Germany
* Correspondence: mona.schmidt@unimedizin-mainz.de; Tel.: +49-6131-17-0

Appendix A

*Search strategies_*

Ovid (Medline)

1. Immunotherapy/ or exp Antineoplastic Agents, Immunological/ or exp Antibodies, Monoclonal, Humanized/ or Immunization/ or Immunotherapy, adoptive/ or Immunotherapy, Active/ or Receptors, Chimeric Antigen/ or Lymphocytes, Tumor-Infiltrating/ or immune checkpoint inhibitors/ or (immunotherap* or immuni#ation* or vaccine-therap* or rna-manipulation* or ((monoclonal or immunological) adj3 antibod* adj3 antineoplastic*) or (Adalimumab or Alemtuzumab or Basiliximab or Certolizumab Pegol or Daclizumab or Ipilimumab or Natalizumab or Nivolumab or Omalizumab or Palivizumab or Ustekinumab or Pembrolizumab or Atezolizumab) or ((checkpoint or pd-1 or pd-l1 or programmed or ctla-4 or cytotoxic-t-lymphocyte) adj3 inhibit*) or ((Tumor or tumour) adj3 (infiltrating or derived) adj3 (cell or lymphocyte*)) or ((chimeric or t-cell) adj3 (receptor* or immunoreceptor*)) or (car-t-cell adj3 therap*)).ab,ti.
2. exp uterine cervical neoplasms/ or (cervi* adj3 (cancer* or carcinom* or neoplas*)).ab,ti.
3. 1 and 2

Web of Science

4. TS=(immunotherap* OR immune?ation* OR vaccine-therap* OR rna-manipulation*)
5. TS=(((monoclonal OR immunological) NEAR/3 (antibod*)) NEAR/3 (antineoplastic*))
6. TS=(Adalimumab or Alemtuzumab or Basiliximab or Certolizumab Pegol or Daclizumab or Ipilimumab or Natalizumab or Nivolumab or Omalizumab or Palivizumab or Ustekinumab or Pembrolizumab or Atezolizumab)
7. TS=((checkpoint OR pd-1 OR pd-l1 OR programmed OR ctla-4 OR cytotoxic-t-lymphocyte) NEAR/3 (inhibit*))
8. TS=((Tumor OR tumour) NEAR/3 (infiltrating OR derived) NEAR/3 (cell OR lymphocyte*))
9. TS=(((chimeric OR t-cell) NEAR/3 (receptor* OR immunoreceptor*)) OR ((car-t-cell) NEAR/3 (therap*)))
10. TS=((cervi*) NEAR/3 (cancer* OR carcinom* OR neoplas*))
11. (((((#1) OR #2) OR #3) OR #4) OR #5) OR #6
12. (#8) AND #7

Cochrane Library

13. MeSH descriptor: [Immunotherapy] this term only
14. MeSH descriptor: [Antineoplastic Agents, Immunological] explode all trees
15. MeSH descriptor: [Antibodies, Monoclonal, Humanized] explode all trees
16. MeSH descriptor: [Immunization] this term only
17. MeSH descriptor: [Immunotherapy, Adoptive] this term only
18. MeSH descriptor: [Immunotherapy, Active] this term only
19. MeSH descriptor: [Receptors, Chimeric Antigen] this term only
20. MeSH descriptor: [Lymphocytes, Tumor-Infiltrating] this term only
21. MeSH descriptor: [Immune Checkpoint Inhibitors] this term only
22. (immunotherap*):ti,ab,kw
23. (immuni?ation*):ti,ab,kw
24. (vaccine-therap*):ti,ab,kw
25. (rna-manipulation*):ti,ab,kw
26. ((monoclonal OR immunological) NEAR/3 antibod* NEAR/3 antineoplastic*):ti,ab,kw
27. (Adalimumab or Alemtuzumab or Basiliximab or Certolizumab Pegol or Daclizumab or Ipilimumab or Natalizumab or Nivolumab or Omalizumab or Palivizumab or Ustekinumab or Pembrolizumab or Atezolizumab):ti,ab,kw
28. ((checkpoint OR pd-1 OR pd-l1 OR programmed OR ctla-4 OR cytotoxic-t-lymphocyte) NEAR/3 inhibit*):ti,ab,kw
29. ((Tumor OR tumour) NEAR/3 (infiltrating OR derived) NEAR/3 cell OR lymphocyte*):ti,ab,kw
30. ((chimeric OR t-cell) NEAR/3 (receptor* OR immunoreceptor*)):ti,ab,kw
31. (car-t-cell NEAR/3 therap*):ti,ab,kw
32.
33. MeSH descriptor: [Uterine Cervical Neoplasms] explode all trees
34. (cervi* NEAR/3 (cancer* OR carcinom* OR neoplas*)):ti,ab,kw
35. {OR #21-#22}
36. #20 AND #23

Table S1. Overview of unspecific immunomodulating therapies. An overview of unscpecific immunomodulating therapies can be found in table S3. However, this cannot be regarded a complete display of existing clinical data on unspecific immunomodulating therapies.

Study/ Author/ Year	Type	Concurrent treatment	Study phase	Number of cervical cancer patients (all patients)	Stage of cervical cancer	Treatment	Survival outcomes	Response rates	Most common adverse events
Wadler et al. 1995 [74]	Interferon α-2b	Prior CHT allowed	II	31	Refractory to conventional treatment Stage I-IV	s.c. 3 times per week	***OS***= 4.5	***ORR***=: 10% ***DCR***= 32.3%	No ***TRD***, hematologic toxicities, fevers, chills, fatigue infection, nausea/vomiting, diarrhea, mucositis,

									neurotoxicity, hepatic/pulmonary toxicities, thrombosis
Wadler et al. 1997 [75]	Interferon α + all-trans retinoic acid	-	II	26	R/M (pretreated)	interferon s.c. 3 times per week + all-trans retinoic acid 3 times per day (continuously or day1-3)	***OS*** = 150 days [81-258]	***ORR*** = 0% ***DCR*** = 34.6%	No ***TRD***, fatigue, anorexia, fevers, chills, dizziness, headache, nausea, dermatologic toxicities (especially xerosis), mild elevation of liver enzymes, mild respiratory symptoms
Wilailak et al. 2003 [76]	Interferon α-2a 3 MU + 13-cis-retinoic acid	CHT (cisplatin + 5-fluorouracil) + RT	I	8	First line Stage IIIB	RT + CHT q1w + interferon s.c. 3 times per week + 13-cis-retinoic acid daily p.o. for 12 weeks	75% alive with CR at 12-48 months (median 24 months), 25% alive with locoregional or distant disease at 18 and 30 months	***CRR*** = 100%	No ***TRD***, hematologic toxicities, nausea/vomiting, elevated liver enzymes, fever, flue-like syndrome, mucositis, diarrhea, peripheral neuropathy
Look et al. 1998 [77]	Interferon α-A2 + isoretinoin	Prior CHT or RT	II	34	R (unresectable)	Daily isoretinoin p.o + daily interferon α-A2 s.c. for 4 weeks per course	***PFS*** in 3 patients with SD 2.8-4.6 months	***ORR*** = 3.8% [0.1-19.6] 3 patients with SD	No ***TRD***, grade 3/4 ***AE*** with hematological toxicities, nausea/vomiting, elevation of liver enzymes or creatinine, neurological symptoms
Braud et al. 2002 [78]	Interferon α + all-trans retinoic acid or 12-cis retinoic acid	CHT (Cisplatin)	II	33	R/M, (pretreated)				
Lippman et al. 1992 [79]	Interferon α-2A plus 13-cis-retinoic acid		II	26	First line Locally advanced	Daily s.c. interferon α-2a + daily oral 13-cis retinoic acid	Not reported	***ORR*** = 50% ***DCR*** = 84.6%	No ***TRD***, dermatitis, conjunctivitiy, fatigue, hypertryglyceridemia
Duenas-Gonzalez et al. 2002 [80]	Leukocyte cytokines (IRX-2)	Surgery (+ adjuvant RT)	I	10	First line IB1-IIA	Neoadjuvant IRX-2, Daily injection of IRX-2 for 10 days + surgery 21d post IRX-2 (+ RT if indicated)	80% disease free at 29-31 months, 1 recurrence at 27 months	Response evaluated during surgery ***ORR*** = 50%	No ***TRD***, no ***AE*** attributed to cytokine treatment
Wadler et al. 2004 [81]	Interleukin-12		II	34	M/R or inoperable (pretreated)	IL-12 i.v. daily for 5 days q3w	***OS*** = 6.5 [5.8-11.5]	***ORR*** = 3% ***DCR*** = 23%	No ***TRD***, hematologic and hepatic toxicities, fever, infection, neurological toxicities, edema, arthralgia/myalgia, fatigue, stomatitis, nausea/vomiting,

									pulmonary and cardiac toxicities
Cappello et al. 1984 [82]	BCG	Surgery	I	45	First line therapy Stage I and II	BCG injections into the portio 21 days prior to surgery	Not reported	Not reported	No ***TRD***, few slightly enlarged lymphnodes
DiSaia et al. 1986 [83]	Corynebacterium parvum	RT	III (RCT)	28	First line therapy Stage IIB-IVA	RT ± C. parvum 4mg/m^2 day 5 and 10 post surgery, then q2w (q4w for 1 year after RT)	No significant differences between groups	Not reported	Related to C. parvum injections: No ***TRD***, chills, fever, lymphopenia, malaise, diarrhea, nausea/vomiting
Mignot et al. 1981 [84]	Corynebacterium parvum	Surgery (+ adjuvant RT)	RCT	58	First line therapy	Neoadjuvant C. parvum injections 10 days prior to surgery	Relapse rate localized tumors: 5% (C. parvum) vs 29% (control); $p<0.05$<br><br>Relapse free interval 13 months ±6.1 (C. parvum) vs 6.5 ±3.9 months (control)	Not reported	No ***TRD***, minor ***AE*** including discomfort, fever
Gall et al. 1978* [85]	Corynebacterium parvum	RT	I	8 (53)	Stage III	RT ± Cornyebacterium parvum 4mg/m^2 day 5 and 10 post surgery, then q2w (q4w for 1 year after RT)	Not reported	Not reported	Overall cohort: No ***TRD***, chills, fever, short-term hypo/hypertension, nausea/vomiting, malaise, diarrhea, minor chest pain, headaches, apprehension, cyanosis, leg cramps
Ahn et al. 2004* [86]	Agaricus blazei Murill Kyowa (ABMK) (mushroom abstract)	CHT (carboplatin + etoposide or taxol	I (RCT)	61 (100)	First line therapy	CHT q3w+ daily p.o. ABMK or placebo	Not reported	Not reported	Improvement of overall physical and emotional conditions
Kikkawa et al. 1993 [87]	OK-432 Streptoccal preparation)	Surgery ± RT	II (RCT)	177	First line Ib and II	Surgery + RT if indicated ± i.d. OK-432 q2d for 5 increasing doses, then q2w for up to 2 years	***OS***= No significant difference between groups<br><br>5y disease free rate= 89% OK-432 group vs 94% control 5y disease free rate for stage II patients= 89%	Not reported	Not reported

							OK-432 group vs. 68% control group (p>0.05)		
Noda et al. 1989 [88]	OK-432 (streptococcal preparation)	RT (±surgery)	II/III? (RCT)	382	First line I-IV	RT ± surgery ± i.d. OK-432 q2d for 5 increasing doses, then q2w for up to 2 years	Stage II cancer= 60m recurrence free rates: 68.6% (OK-432) vs 50.7 (control) p<0.05 60m survival rate= 72.4% (OK-432) vs 56.5% (p=0.07)<br><br>Stage III cancer 60m recurrence free rates= 36-7% (OK-432) vs 35.6% (control) (p<0.05) 60m survival rates= 44.7 (OK-432) vs 40.2% (control) p>0.05<br><br>Sig. prolonged survival time in patients undergoing surgery for stage II cancer with OK-432	Not reported	Not reported
Okamura et al. 1989 [89]	OK-432 (streptococcal preparation)	Unclear	(RCT)	387	Primary cervical cancer, stratified by previous surgery	OK-432 vs unknown control treatment	***Recurrence free rates***= 12m 84.7% (OK-432) vs 4.7%(control) 24m= 75.9% vs 71.8% 36m= 71.9% vs 58.6% (p<0.05) Difference not sig. in stage III subgroup	Not reported	Not reported
Kucera et al. 1982 [90]	Vitamin A	RT	I/II (RCT)	42	First line Inoperable stage III	RT ± vitamin A palmitate p.o.5 days/week during the first 4 weeks of RT	***1yPFS=*** 76.2% (vitamin A group) vs 61.2%	Not reported	Vitamin A related ***AE***: dermatitis, stomatitis
Mallmann et al. 1989* [91]	Thymopentin	Surgery or RT depending on stage		34 (74)	First line Stage I-IV	Surgery or RT depending on stage ± thymopentin 100mg/die for 2 weeks, then 100mg 3x/week for 4 weeks	Cervical carcinoma: reported only in graphs, no statistical analysis performed, graphically no great differences	Not reported	Not reported

Noda et al. 1992 [92]	Sizofiran	RT	II? (RCT)	292	First line Stage II-III	RT± i.m. sizofiran 40mg initially, then 20mg/week until 1 week post RT	Not reported	**CRR=** 83% (77.5-88.5; sizofiran) vs 69.6% (59.6-79.5; control), p<0.05	No ***TRD***, no ***SAE***, mild injection site reactions, rash, nausea/vomiting, increased liver enzymes
Okamura et al. 1989 [89]	Sizofiran	RT	RCT		First line, Stage II-III	RT± i.m. sizofiran 40mg once or twice per week	Reported as graphs, Sig. longer time to recurrence overall as well as stage II patients, not in stage III with sizofiran<br><br>6 months survival curves sig different overall and in stage II patients with sizofiran	Not reported	Not reported
Noda et al. 2006A [93]	Z-100 (extract of mycobacterium tuberculosis strain Aoyama B)	RT	II (RCT)	116	First line Stage IIIB	RT+ s.c. Z-100 twice per week during RT followed by q1w (3 different doses tested)	***5y OS***= 40μg: 47.4% [30.6-64.2] 20μg: 59.6% [43.8-75.4% 2μg: 59.2% [42.7-75.7] P=0.598	**ORR**= 83.6%<br><br>**ORR** at 40μg= 94.3% **ORR** at 20μg= 84.6% **ORR** at 2μg= 72.2% P=0.006	No ***TRD***, no ***SAE***; fever, injection site reactions, rash, elevated liver enzymes, headache
Noda et al. 2006B [94]	Z-100 (extract of mycobacterium tuberculosis strain Aoyama B)	RT (+ CHT if indicated)	III (RCT)	221	First line Stage IIIB	RT+ s.c. Z-100 twice per week during RT followed by q2w (2 different doses tested)	***PFS***= 0.2μg: 80 months [39-NR] 40μg: 30 months [19-not reported] ***OS***= 0.2μg NR [57-NR] 40μg: 44 months [38-62], p<0.05<br><br>***5y OS***= 0.2μg: 58.2% [48.7-67.7] 40μg: 41.5% [31.7-51.3], p<0.05 ***HR***= 0.67 [0.46-0.98]	**ORR**= 0.2μg: 93.5% 40μg: 91.5%, p>0.05	No **TRD**, no sig. differences between grade 3/4 ***AE*** both groups, muscolo-skeletal disorders, gastrointestinal disorders, urinary system disorders, general disorders, respiratory issues, cardiovascular disorders
Sugiyama et al. 2014 [95]	Z-100 (extract of mycobacterium tuberculosis strain	RT (+CHT if indicated)	III (RCT)	249	First line Stage IIB-IVA	RT + Z-100 0.2μg vs placebo twice per week during RT, then q2w	Trial stopped prematurely due to high survival rates	**ORR=** 99% (Z-100) vs 99% (placebo), **CRR=** 68.3% (Z-100) vs 54.5% (placebo)	No ***TRD***, similar ***AE*** between both groups, leukopenia higher in placebo group, constipation/diarrhea, nauseas/vomiting,

	Aoyama B)						***5yOS*=** 75.7% (Z-100) vs 65.8%, p=0.07<br>***OS*** in stage III patients sign. prolonged with Z-100 (HR 0.51 [0.28-0.94]		dermatitis, cystitis, headache, anorexia, fatigue, radiation-associated pain, anemia
Okawa et al. 1989 [96]	LC9018 (prepared from Lactobacil lus casei)	RT	II (RCT)	61	First line Stage IIB or III	RT± i.d. or s.c. LC9018 once or twice per week	***3yOS*=** 73.1% (LC9018) vs 60.9 (control), p>0.05	**ORR=** 100% in both groups post RT, sig. quicker response in LC9018 group<br><br>**CR=** 69.2% (LC9018) vs 56.5% (control), p>0.05<br><br>**CR** by administration route: 88.2% (i.d.) vs 33.3% (s.c.), p<0.05	No ***TRD***, fever, local injection site reactions, anorexia, diarrhea
Okawa et al. 1993 [97]	LC9018 (Prepared from Lactobacil lus casei)	RT	III (RCT)	213	First line Stage IIIB	RT± i.d. LC9018 once or twice per week during RT, then once per week or month for two years	***4y recurrence rate*=** 33.5% (LC9018) vs 51.5% (control), p<0.05<br><br>***4yOS*=** 69.2% (LC9018) vs 46.2% (control), p<0.05	**ORR=** 92.6% (LC9018) vs 89.7% (control), p=0.094<br><br>**CRR=** 53.7 % (LC9018) vs 42.1% (control)	Injection site reaction, mild fever (LC9018), diarrhea, anorexia, less neutropenia in the LC9018 group, liver enzyme elevations, anemia

AE: Adverse events; CHT: chemotherapy; CRR: complete response rate; DCR: disease control rate; HR= hazard ratio, i.d.: intra-dermally; i.v.: intravenously; ORR: objective response rate; OS: overall survival; p.o.: per os; PFS: progression-free survival; RCT: randomized controlled trial; RT= radiotherapy, s.c.: subcutaneously; SAE: serious adverse events; TRD: treatment-related deaths, Xy OS: X-year overall survival

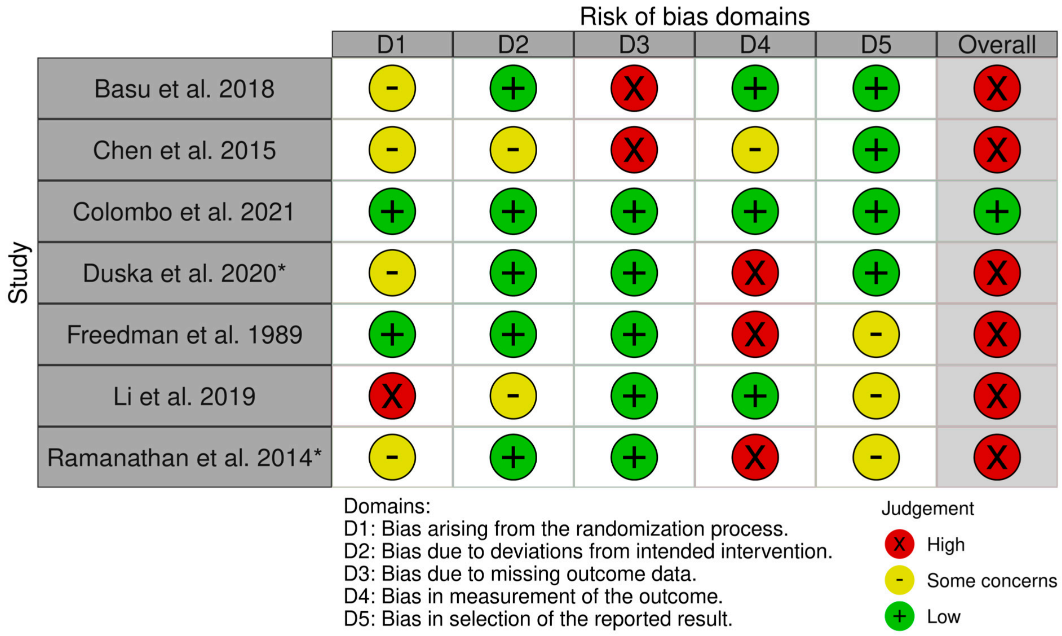

Figure S1. Risk of bias assessment of included randomized controlled trials – Individual trial judgements. * Trials were assessed for safety.

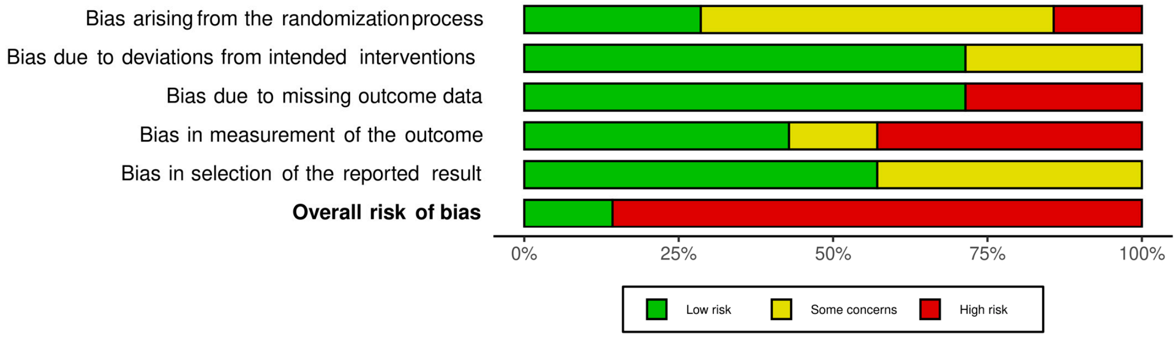

Figure S2. Risk of bias assessment of included randomized controlled trials – Collective risk of bias assessments.

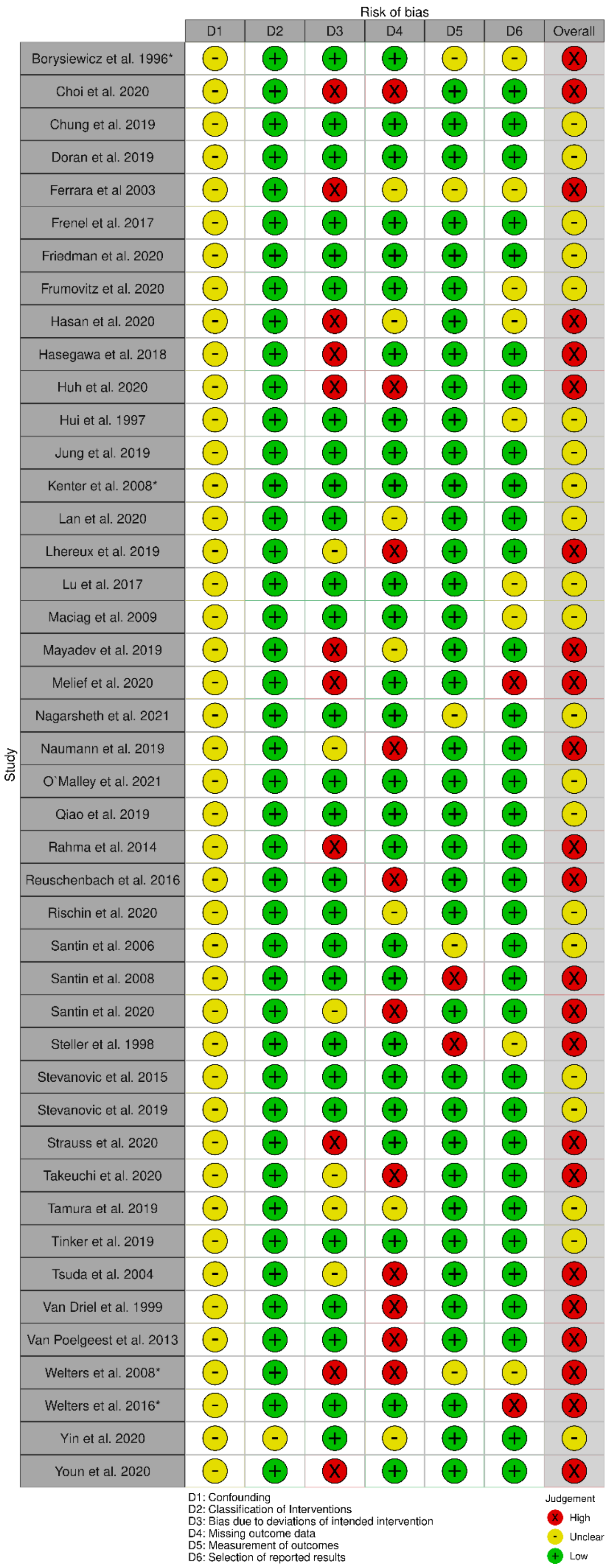

Figure S3. Risk of bias assessment of included single arm cohort trials – Individual trial judgements. * Trials were assessed for safety.

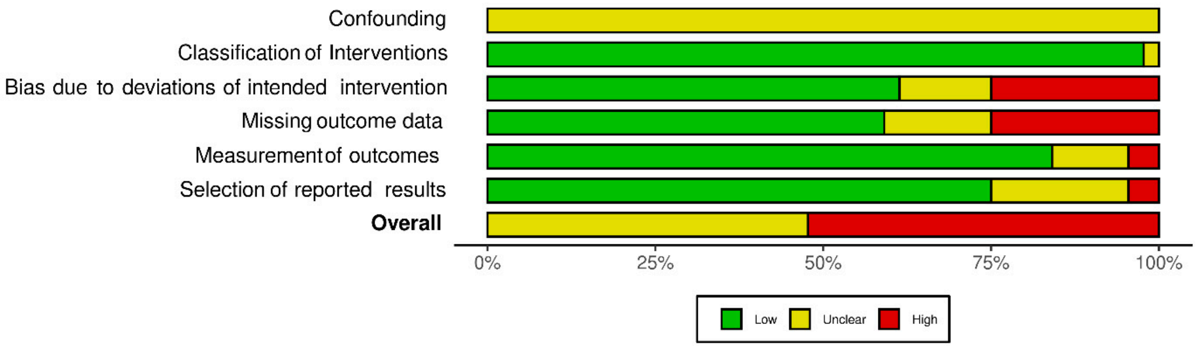

Figure S4. Risk of bias assessment of included single arm cohort trials – Collective risk of bias assessments.
